# A Well‐Defined Anionic Dicopper(I) Monohydride Complex that Reacts like a Cluster**

**DOI:** 10.1002/anie.202202318

**Published:** 2022-05-09

**Authors:** Roel L. M. Bienenmann, Alexandra J. Schanz, Pascale L. Ooms, Martin Lutz, Daniël L. J. Broere

**Affiliations:** ^1^ Organic Chemistry and Catalysis Debye Institute for Nanomaterials Science Faculty of Science Utrecht University Universiteitsweg 99 3584 CG Utrecht The Netherlands; ^2^ Structural Biochemistry Bijvoet Centre for Biomolecular Research Faculty of Science Utrecht University Universiteitsweg 99 3584 CG Utrecht The Netherlands

**Keywords:** Copper Hydrides, Dinuclear Complexes, Expanded Pincer, Homogeneous Catalysis, Hydrosilylation

## Abstract

Low‐nuclearity copper hydrides are rare and few well‐defined dicopper hydrides have been reported. Herein, we describe the first example of a structurally characterized anionic dicopper hydride complex. This complex does not display typical reactivity associated with low‐nuclearity copper hydrides, such as alcoholysis or insertion reactions. Instead, its stoichiometric and catalytic reactivity is akin to that of copper hydride clusters. The distinct reactivity is ascribed to the robust dinuclear core that is bound tightly within the dinucleating ligand scaffold.

## Graphical abstract

A rare, well‐defined dicopper hydride (Cu_2_H) complex is reported, that displays divergent reactivity from known Cu_2_Hs. Using a combined experimental and computational approach, we ascribe the cluster‐like reactivity to the rigid dinucleating nature of the expanded pincer ligand, which prevents the formation of mononuclear CuHs in solution.

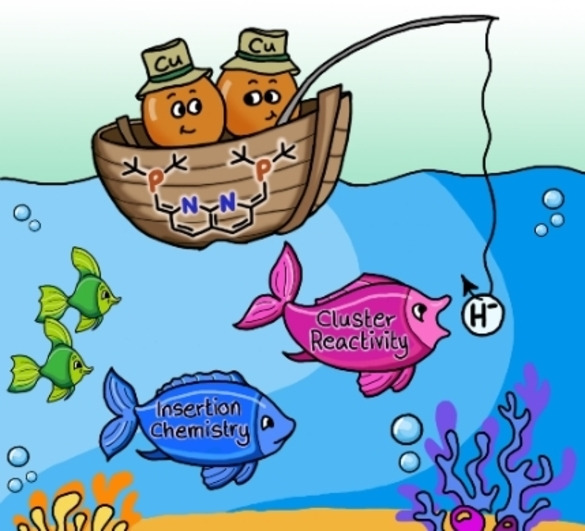

Copper hydrides (CuHs) have found widespread use in homogeneous reduction catalysis.^[1]^ This started with seminal work by Stryker and co‐workers, who found that the hexameric CuH cluster [Cu(PPh_3_)H]_6_ (Stryker's reagent) catalyzes the selective reduction of α,β‐unsaturated ketones under high H_2_ pressure.^[2]^ Following this report, it was found that hydrosilanes are excellent terminal reductants for this reaction, allowing for milder reaction conditions.[^3^, ^4^, ^5^] Since then, numerous CuH complexes have been reported for the reduction of carbonyl compounds through hydrosilylation showing high activity, enantioselectivity or functional group tolerance.[^1^, ^4^] Despite their wide usage, the identity and aggregation state of the active species is often unclear, due to the typical in situ preparation of the catalyst.

In recent years, there has been increasing attention for the application of well‐defined multinuclear complexes in catalysis.[^6^, ^7^, ^8^, ^9^] In these systems, the cooperative function of multiple metal centers enables distinct reactivity from their mononuclear counterparts.^[10]^ Towards this end, our group has been working on dinuclear copper(I) complexes supported by the PNNP expanded pincer ligand.^[11]^ This ligand can bind two Cu(I) atoms in close proximity, and can be deprotonated once or twice concomitant with partial or full dearomatization of the naphthyridine core.[^11^, ^12^] The latter enabled the synthesis of tetracopper dihydride complex **A** (Scheme 1) through bifunctional activation of hydrogen gas.^[11]^ Recently, Tilley and co‐workers reported penta‐ and trinuclear cationic CuH complexes supported by a naphthyridine based ligand (Scheme 1, **B**). These complexes were found to catalyze formic acid dehydrogenation, which is uncommon for CuHs.^[13]^ A notable similarity in all reported naphthyridine‐based CuHs is the strong driving force for aggregation of three or more metal centers to stabilize the copper hydride core.[^11^, ^13^]

**Scheme 1:**
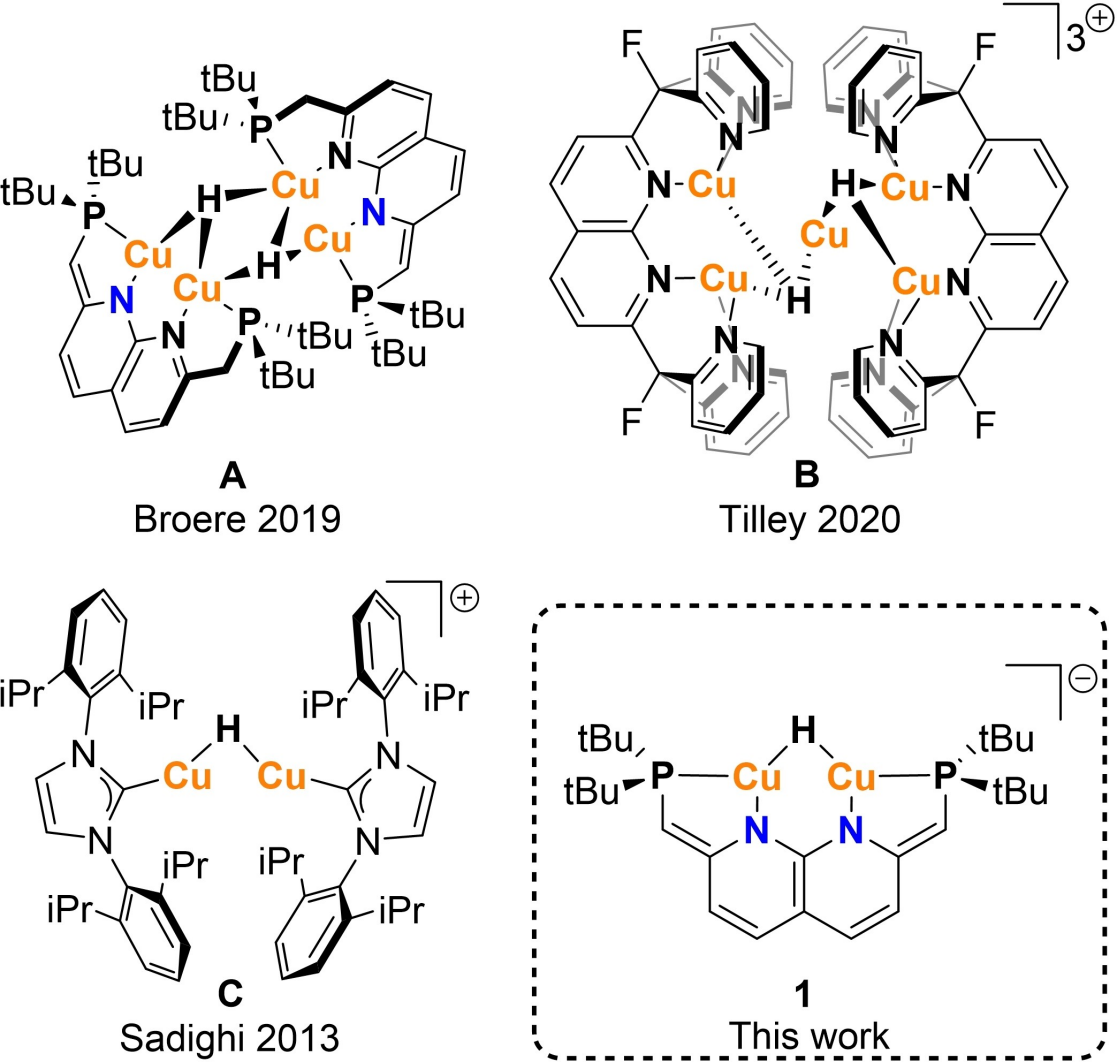
Previous work on related copper hydride complexes.

The tendency of CuHs to form oligomers and other polynuclear complexes through electron deficient multicenter two‐electron bonding has resulted in the structural characterization of a plethora of CuH clusters.[^14^, ^15^, ^16^, ^17^] In contrast, well‐defined low‐nuclearity CuHs are rare and especially mono‐ and dinuclear CuHs are only sporadically isolated and structurally characterized (see Table S1).[^18^, ^19^, ^20^, ^21^, ^22^, ^23^, ^24^, ^25^, ^26^, ^27^, ^28^] In 2013 Sadighi and co‐workers showed that bulky N‐heterocyclic carbene (NHC) ligands can be exploited for the stabilization of dinuclear CuHs (Scheme 1, **C**).[^20^, ^22^] Subsequently, numerous CuH complexes bearing sterically encumbering NHC ligands have been reported and employed as catalysts for the functionalization of unsaturated organic substrates.[^23^, ^26^, ^27^, ^28^, ^29^, ^30^] However, the instability of low‐nuclearity CuHs and their fluxional behavior in solution have hampered mechanistic studies of these transformations. Very recently, Bullock and co‐workers reported detailed mechanistic studies on the insertion chemistry of [(NHC)Cu(μ‐H)]_2_ complexes showing the importance of CuH monomerization to enable insertion of unsaturated substrates.[^27^, ^30^] However, investigations into the inherent reactivity of dinuclear monohydrides themselves are absent due to the lack of robust systems that do not change aggregation state in solution.

In this work, we report the synthesis of a well‐defined anionic dicopper monohydride complex (**1**) that retains its dinuclear nature in solution. Despite its low nuclearity and steric accessibility of the hydride, we show that complex **1** is stable and lacks the distinct hydridic reactivity that is associated with low‐nuclearity CuHs. Instead, we demonstrate that its stoichiometric and catalytic reactivity is akin to CuH clusters such as Stryker's reagent, despite containing only two copper centers.

The previously reported complex **A** does not display catalytic activity in the hydrosilylation of aldehydes or ketones. We reason that the dimeric nature, which is stabilized by the electron deficient 4‐center 2‐electon bonding in the Cu_4_H_2_ core, renders the hydrides sterically inaccessible (Figure S1). Hence, we hypothesized that preventing this dimerization would lead to increased reactivity of the hydride core. It was envisioned that deprotonation of the PNNP methylene linkers in complex **A** could decrease the driving force for dimer formation due to a combination of increased electron density on the dicopper core and coulombic repulsion. Treatment of complex **A** with benzyl potassium, which we previously utilized to access the fully dearomatized PNNP ligand on dicopper(I),^[11]^ resulted in the formation of intractable mixtures. The addition of KOtBu to a THF solution of **A** does not lead to any deprotonation of **A** according to NMR analysis. However, subsequent addition of 18‐crown‐6, which sequesters the potassium cation to increase the basicity,^[31]^ results in full conversion of **A** to complex **1** (Scheme 2).

**Scheme 2:**
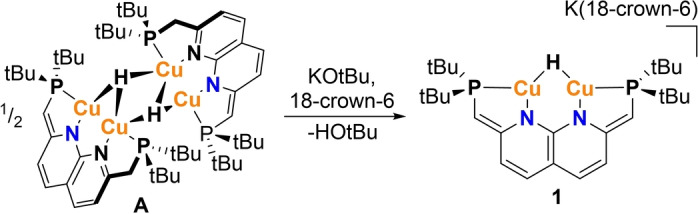
Synthesis of dinuclear copper(I) hydride complex **1**.

Complex **1** was isolated as an extremely air and moisture sensitive orange powder in 75 % yield. The ^31^P{^1^H}‐NMR spectrum of **1** in THF‐d_8_ at 25 °C features a single resonance at *δ*=20.5 ppm, showing both phosphorous atoms are magnetically equivalent. The ^1^H‐NMR spectrum shows two doublets at *δ*=6.12 ppm (^3^ *J*_H,H_=8.3 Hz) and 5.55 ppm (^3^ *J*_H,H_=8.3 Hz) corresponding to the naphthyridine backbone. A singlet at *δ*=3.22 ppm corresponding to the methine linkers integrates equally to each backbone signal. These features in the NMR spectra are characteristic for a *C*_2*v*_ symmetric, fully dearomatized PNNP ligand.^[11]^ The hydride ligand is magnetically coupled to both phosphorous nuclei and is found as a triplet at *δ*=0.89 ppm (^3^ *J*_H,P_=25.3 Hz). The identity of this hydride peak was confirmed by ^2^H‐NMR of the deuteride analogue of **1** (**1D**, see Supporting Information). The structure of complex **1** was revealed with single crystal X‐ray crystallography, showing a monomeric dicopper hydride (Figure 1) as opposed to dimeric complex **A**.^[32]^ Structurally characterized dicopper hydride complexes are rare and all reported examples are neutral or cationic (Table S1). To the best of our knowledge, complex **1** is the first structurally characterized anionic dicopper hydride complex.

**Figure 1:**
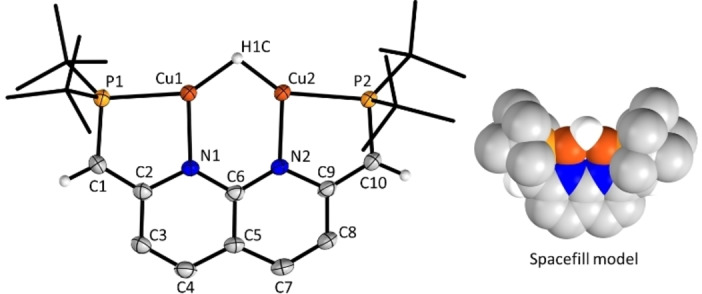
The structure of **1** determined by X‐ray diffraction. Displacement ellipsoids (left) are displayed at 50 % probability, t‐Bu groups are depicted as wireframe, and the K (18‐crown‐6) counterion and two THF molecules are omitted for clarity. Spacefill spheres (right) are shown at the Van der Waals radius.

Unlike the hydrides in complex **A** (Figure S1), the hydride in **1** is remarkably exposed (Figure 1). The Cu⋅⋅⋅Cu distance (2.4229(7) Å) in **1** is shorter than the Cu⋅⋅⋅Cu distances in **A** (2.4778(5)–3.4144(6) Å),^[11]^ which is in line with the trend observed for the dearomatization of the analogous mesityl complexes.^[12]^ Dearomatization of the backbone is evident from the short C1–C2 and C9–C10 bond lengths (1.387(3) and 1.386(3) Å, respectively) which are consistent with localized C=C double bonds. This dearomatization leads to a rigid backbone structure in which the methine linkers are in plane with the naphthyridine backbone (P1−C1−C2−N1=2.7(3)°). The hydride ligand was located in the Fourier difference map and DFT calculations (see Supporting Information) indicate that it is bound symmetrically between the two copper centers, in line with the *C*_2*v*_ symmetry of the complex observed in solution.

Low‐nuclearity copper hydrides typically undergo protonolysis in the presence of alcohols or other weak acids to form H_2_ gas.[^22^, ^26^] In contrast, complex **1** is stable towards *tert*‐butanol (formed in its synthesis). Moreover, adding a strong acid such as HBArF_24_ ^[33]^ to complex **1** does not lead to the formation of H_2_ gas, but results in protonation of the ligand backbone to give complex **A** (Scheme 3). This type of reactivity is in line with the DFT calculated HOMO of **1** that is located mainly on the methine linkers, rather than on the hydride ligand (Figure S29). Similarly, exposure of THF solutions of **1** to ambient air leads to immediate formation of complex **A** together with several unidentified side products based on NMR analysis. Notably, reacting **1** at 80 °C with 4‐fluorophenylacetylene, which is a weak C−H acid, does result in the formation of H_2_ gas and the corresponding acetylide complex **2** (Scheme 3). However, monitoring this reaction with ^1^H‐NMR spectroscopy showed that at room temperature initially **A** is formed. When the mixture is heated, formation of **2** and H_2_ is observed. The intermediacy of **A** in this reaction, rather than direct reaction of the hydride in **1** is in line with the lack of hydridic reactivity of this complex. Similar ligand‐centered reactivity is observed upon exposure of **1** to an atmosphere of CO_2_. Initial formation of a mixture of unstable intermediates containing partially dearomatized ligand backbones is observed (see Supporting Information for discussion), and these subsequently convert into **A**, an unidentified species and potassium formate.

**Scheme 3:**
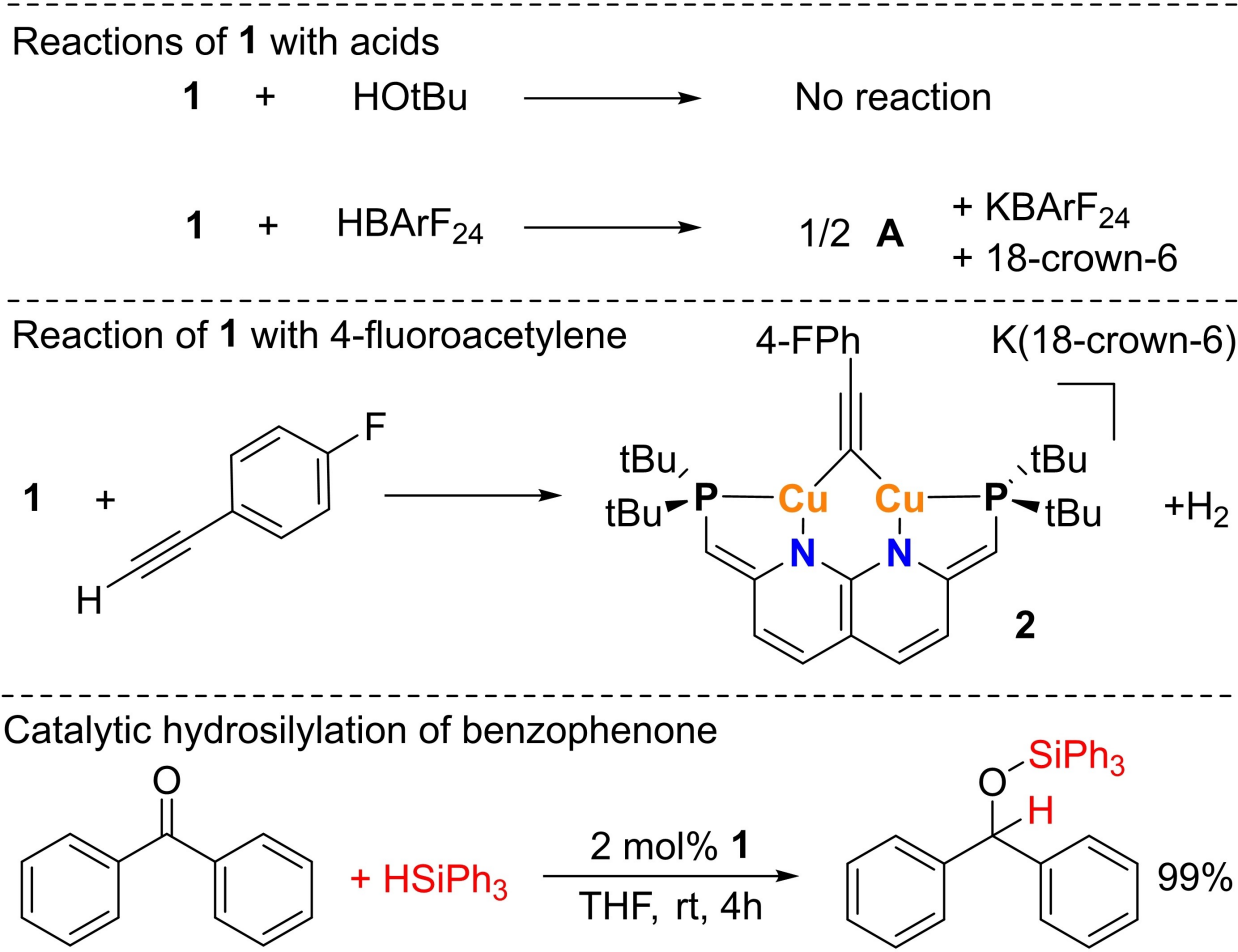
The reaction of complex **1** with acids (top) and alkynes (middle). The catalytic hydrosilylation of benzophenone with **1**.

Dicopper hydrides are known be susceptible to insertion reactions of unsaturated substrates into the Cu−H bond, hence we explored such reactivity for **1**.[^19^, ^20^, ^22^, ^23^, ^26^, ^27^, ^28^, ^30^] As discussed above, the addition of a terminal alkyne did not lead to an insertion into the Cu−H bond, contrary to what is observed for other dicopper hydrides.[^20^, ^22^, ^24^, ^27^] Similarly, the addition of other unsaturated substrates to **1** (alkenes, internal alkynes, ketones, nitriles and imines) did not lead to the corresponding insertion products despite the lack of steric encumbrance around the hydride (Figure 1). Altogether, it is evident that **1** reacts distinctly different from reported dicopper hydrides. CuH clusters, including Stryker's reagent are well known for their catalytic activity in the hydrosilylation of ketones and aldehydes.[^1^, ^4^] Similarly, we found that **1** catalyzes these transformations at room temperature in THF. Contrary to Stryker's reagent, **1** is also able to hydrosilylate bulky ketones such as benzophenone with bulky silanes like triphenyl silane (Scheme 3) under these conditions (see Supporting Information).

To gain more insights into the principles underlying the distinct reactivity of **1**, we probed the properties of this complex with DFT calculations. Natural Bonding Orbital (NBO) analysis shows that the hydride is bound via an open 3‐center 2‐electron (3c–2e) bond, polarized towards the hydride. Interestingly, natural population analysis shows that the charge of the hydride ligand in **1** has decreased slightly compared to the hydrides in complex **A** (−0.04 e^−^), despite the overall anionic charge on complex **1** (Scheme 4, bottom). To investigate the origin of this, we considered that the deprotonation of **A** to form **1** can theoretically be split into two processes: the monomerization and the deprotonation itself. To assess the influence of each of these on the charge distribution in the molecule, we calculated the stepwise change in electron density upon monomerization to the theoretical **A_mono_**, and the subsequent change in electron density upon deprotonation to **1** (Scheme 4, middle). This revealed that the decrease in charge on the hydride ligand is due to the monomerization, rather than the deprotonation itself. This can be rationalized by considering that the driving force for the dimerization is the delocalization of electron density by electron deficient bonding via open multi‐center 2‐electon bonds.^[14]^ Due to the lack of direct covalent bonding between the d^10^ Cu(I) centers, this delocalization of the electron density increases the occupancy of the hydride 1 s orbital. Hence, in higher nuclearity multi‐center bonding, there will be more electron density on the hydride as we see in complex **A** as compared to **A_mono_**_._ The natural population analysis also shows that the increase in electron density upon deprotonation (i.e. going from **A_mono_** to **1**) is mainly localized on the naphthyridine backbone, and this is also evident in the highest occupied molecular orbital (HOMO) of **1**, which is situated on the naphthyridine backbone (Figure S25).

**Scheme 4:**
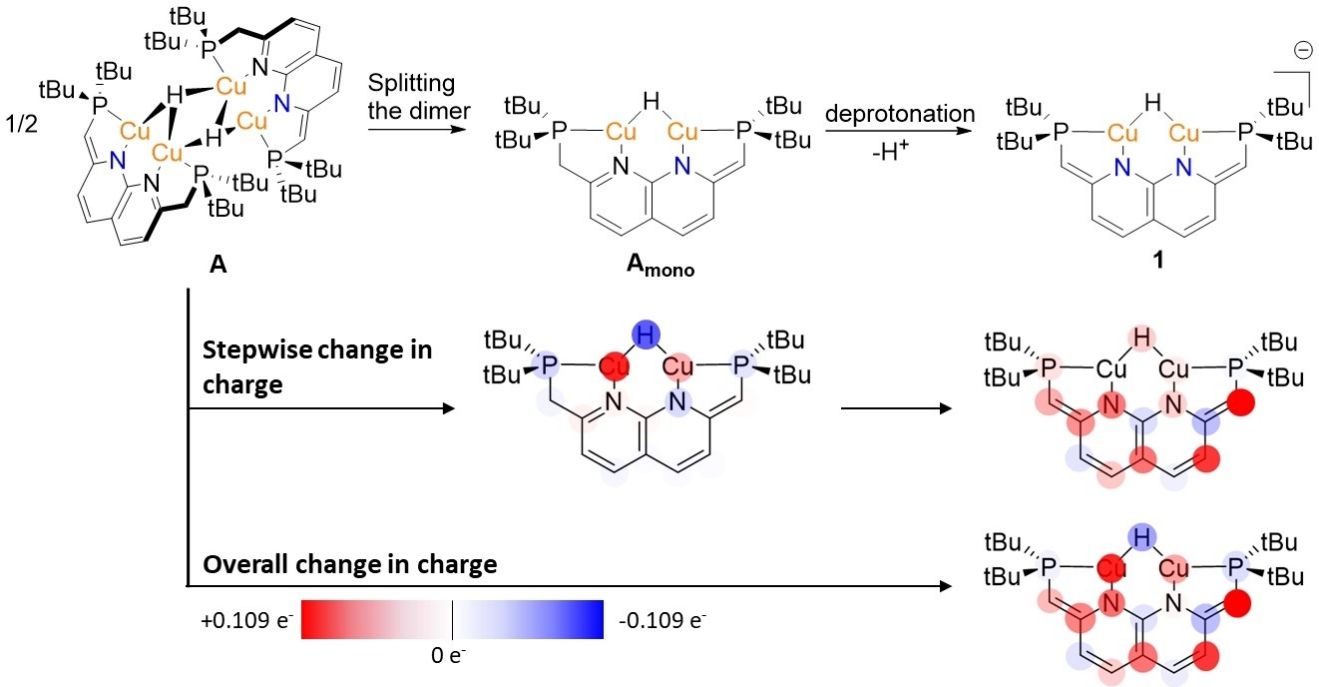
The change in electron density (natural population) upon deprotonation of **A** forming **1**. Blue indicates a depletion of charge and red an increase. The intensity scales with the magnitude of the relative in‐ or decrease with respect to the species before each arrow.

To explain the distinct reactivity of **1** with respect to other dicopper hydrides we also performed NBO calculations on the cationic dicopper hydride reported by Sadighi and co‐workers (Scheme 1, **C**) for fair comparison of the electronic structure (See Supporting Information). These calculations show similar open type 3c–2e bonding in both complexes, where the Cu⋅⋅⋅Cu interaction is mediated through the bridging hydride. This indicates that the distinct reactivity of **1** is not due a lack of hydridic character, especially given that the calculations show that the Cu_2_−H bond in **1** is even more polarized towards H than in Sadighi's complex.^[22]^

Given that **1** was found to not react as a typical hydride, we were interested to discover if the catalytic hydrosilylation proceeds via a hydride insertion pathway as has been shown for low‐nuclearity Cu−H species.^[30]^ Hence, we performed a hydrosilylation reaction using stoichiometric amounts of Ph_2_SiD_2_, **1** and benzophenone, which yielded the corresponding silyl ether product containing 94 % deuterium on the benzylic position (Scheme 5, top). This experiment shows that the hydride in **1** is not incorporated into the silyl ether product. The 6 % of silyl ether in which there is a C*H* present is the result of H/D exchange of complex **1** with Ph_2_SiD_2_ to give **1D** and Ph_2_SiHD (Scheme 5, middle). This reaction happens at a slower, yet non‐negligible rate under the conditions of the experiment. Additionally, **1** also catalyzes siloxane scrambling (Scheme 5, bottom), which is the reason that mostly SiPh_2_(OCHPh_2_)_2_ is observed instead of HSiPh_2_(OCHPh_2_) (0.44 : 0.12). The same product ratio is also observed when **1** is reacted with pure HSiPh_2_(OCHPh_2_).

**Scheme 5:**
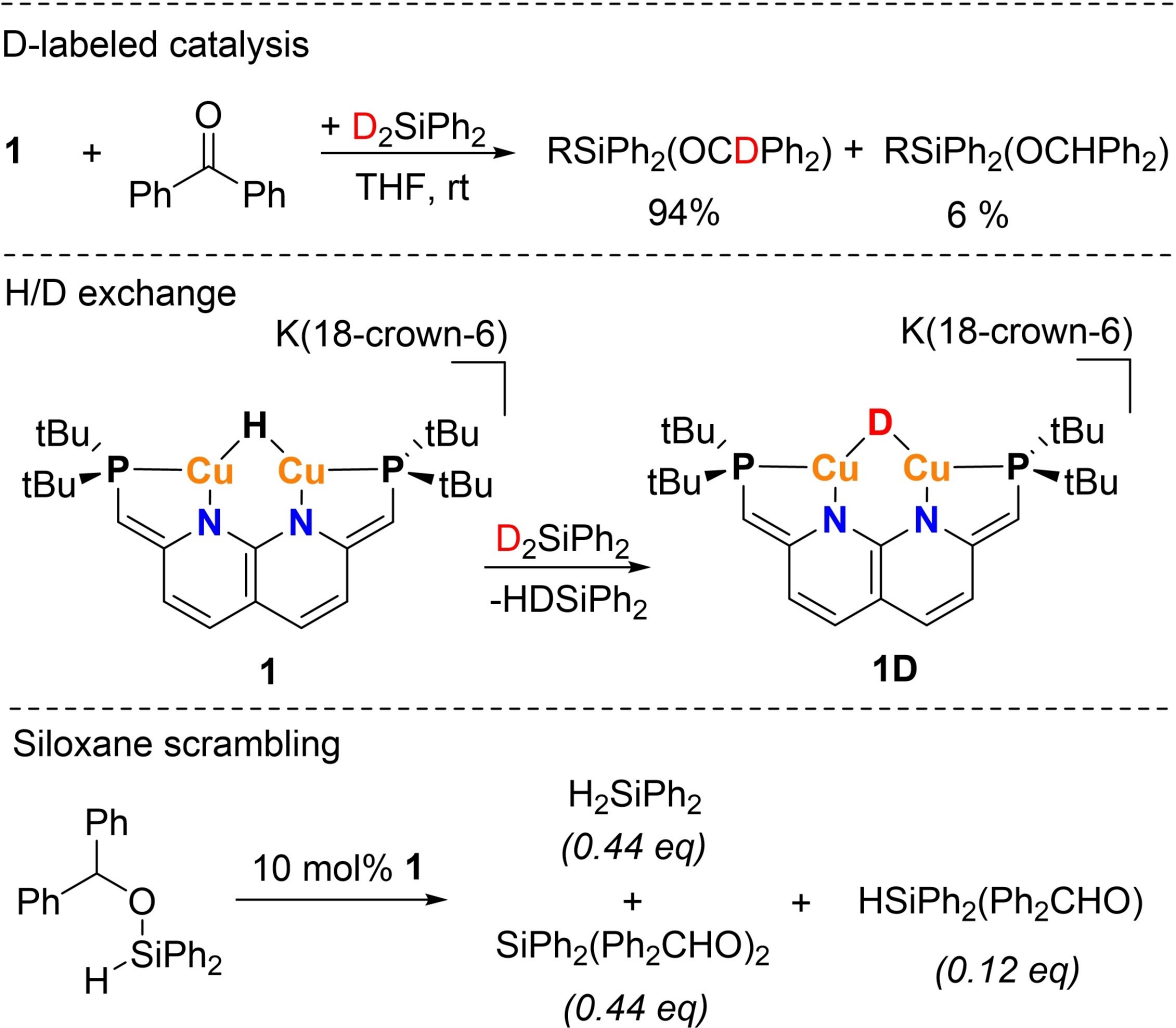
The catalytic hydrosilylation of benzophenone with **1** with D_2_SiPh_2_ (R=H/D or OC(H/D)Ph_2_) (top). The H/D exchange of **1** to **1D** with a deuterated silane (middle). The siloxane scrambling catalyzed by **1** (bottom).

Stryker's reagent has been shown to react stoichiometrically with benzaldehyde to form the insertion product, and is catalytically able to hydrosilylate it.^[34]^ Therefore it was assumed that for ketones, Stryker's reagent reacts through an insertion mechanism in hydrosilylation catalysis. However, Nikonov and co‐workers showed by isotopic labeling that the hydride in Stryker's reagent is not incorporated into the final product, similarly to what we observed for complex **1**.^[35]^ It is important to note that computational studies into the mechanism for copper hydride catalyzed ketone hydrosilylation assume the presence of mononuclear species in solution, in which case the insertion pathway is viable.^[36]^ The aggregation state of such species in solution is, however, often unclear.

Comparing the reactivity of **1** with that of known CuHs, the reactivity of **1** is akin to that observed for CuH clusters such as Stryker's reagent, which tolerates alcohols and does not follow a hydride insertion pathway in ketone hydrosilylation.[^1^, ^35^] We reason that this cluster‐like behavior is due to the presence of the rigid dinucleating ligand in **1**. The absence of such a ligand in reported dicopper hydrides enables dissociation into reactive mononuclear CuH species. This implies that the rich insertion chemistry observed for low‐nuclearity CuHs is not inherent to their low nuclearity, but rather to their ability to dissociate into mononuclear CuH species.[^26^, ^27^, ^30^, ^37^] We hypothesize that such coordinatively unsaturated species are needed for pre‐coordination of the unsaturated substrate enabling hydride insertion.

In conclusion, we have synthesized the first structurally characterized anionic dicopper monohydride complex, **1**. Despite its anionic charge, **1** does not display the strongly hydridic reactivity that is observed for cationic and neutral dicopper hydrides supported by mononucleating ligands. We reason that the rigid PNNP ligand prevents the formation of mononuclear CuH species that are needed for hydride insertion pathways. As a result, **1** reacts like a copper hydride cluster despite its low nuclearity. This work shows that there is more to the distinct reactivity of dicopper hydrides than low‐nuclearity alone. Moreover, it provides a platform that enables studying the inherent reactivity of dinuclear copper hydrides themselves. Future efforts in our laboratories will focus on using this platform to shed new light on the mechanisms of chemical transformations that are catalyzed by copper hydride clusters.

## Supporting Information

The experimental procedures and data supporting the work in this paper is available in the supporting materials. NMR and computational data files can be obtained from the 4TU database under DOI: 10.4121/19145936.

## Conflict of interest

The authors declare no conflict of interest.

## Supporting information

As a service to our authors and readers, this journal provides supporting information supplied by the authors. Such materials are peer reviewed and may be re‐organized for online delivery, but are not copy‐edited or typeset. Technical support issues arising from supporting information (other than missing files) should be addressed to the authors.

Supporting Information

## Data Availability

The data that support the findings of this study are openly available in 4TU.ResearchData at https://doi.org/10.4121/19145936.v1.

## References

[1] C. Deutsch , N. Krause , B. H. Lipshutz , Chem. Rev. 2008, 108, 2916–2927. 18616323 10.1021/cr0684321

[2] W. S. Mahoney , D. M. Brestensky , J. M. Stryker , J. Am. Chem. Soc. 1988, 110, 291–293.

[3] B. H. Lipshutz , W. Chrisman , K. Noson , P. Papa , J. A. Sclafani , R. W. Vivian , J. M. Keith , Tetrahedron 2000, 56, 2779–2788.

[4] S. Díez-González , S. P. Nolan , Acc. Chem. Res. 2008, 41, 349–358. 18281951 10.1021/ar7001655

[5] B. H. Lipshutz , J. Keith , P. Papa , R. Vivian , Tetrahedron Lett. 1998, 39, 4627–4630.

[6] D. R. Pye , N. P. Mankad , Chem. Sci. 2017, 8, 1705–1718. 29780450 10.1039/c6sc05556g PMC5933431

[7] I. G. Powers , C. Uyeda , ACS Catal. 2017, 7, 936–958.

[8] P. Kalck , Topics in Organometallic Chemistry Homo- and Heterobimetallic Complexes in Catalysis, Springer, Heidelberg, 2016.

[9] J. Campos , Nat. Chem. Rev. 2020, 4, 696–702. 10.1038/s41570-020-00226-5 37127975

[10] Y.-Y. Zhou , C. Uyeda , Science 2019, 363, 857–862. 30792299 10.1126/science.aau0364 PMC6467289

[11] E. Kounalis , M. Lutz , D. L. J. Broere , Chem. Eur. J. 2019, 25, 13280–13284. 31424132 10.1002/chem.201903724 PMC6856846

[12] E. Kounalis , M. Lutz , D. L. J. Broere , Organometallics 2020, 39, 585–592.

[13] A. N. Desnoyer , A. Nicolay , M. S. Ziegler , N. A. Torquato , T. D. Tilley , Angew. Chem. Int. Ed. 2020, 59, 12769–12773; 10.1002/anie.202004346 32372506

[14] J. T. B. H. Jastrzebski , G. Kooten in Modern Organocopper Chemistry (Ed.: N. Krause ), Wiley-VCH, Weinheim, 2002.

[15] C. Sun , B. K. Teo , C. Deng , J. Lin , G. G. Luo , C. H. Tung , D. Sun , Coord. Chem. Rev. 2021, 427, 213576.

[16] R. S. Dhayal , W. E. Van Zyl , C. W. Liu , Acc. Chem. Res. 2016, 49, 86–95. 26696469 10.1021/acs.accounts.5b00375

[17] H. Shen , L. Wang , O. López-Estrada , C. Hu , Q. Wu , D. Cao , S. Malola , B. K. Teo , H. Häkkinen , N. Zheng , Nano Res. 2021, 14, 3303–3308.

[18] E. A. Romero , P. M. Olsen , R. Jazzar , M. Soleilhavoup , M. Gembicky , G. Bertrand , Angew. Chem. Int. Ed. 2017, 56, 4024–4027; 10.1002/anie.201700858 28251762

[19] G. V. Goeden , J. C. Huffman , K. G. Caulton , Inorg. Chem. 1986, 25, 2484–2485.

[20] N. P. Mankad , D. S. Laitar , J. P. Sadighi , Organometallics 2004, 23, 3369–3371.

[21] G. D. Frey , B. Donnadieu , M. Soleilhavoup , G. Bertrand , Chem. Asian J. 2011, 6, 402–405. 20945453 10.1002/asia.201000576 PMC3117327

[22] C. M. Wyss , B. K. Tate , J. Bacsa , T. G. Gray , J. P. Sadighi , Angew. Chem. Int. Ed. 2013, 52, 12920–12923; 10.1002/anie.201306736 24132865

[23] K. Nakamae , B. Kure , T. Nakajima , Y. Ura , T. Tanase , Chem. Asian J. 2014, 9, 3106–3110. 25204731 10.1002/asia.201402900

[24] A. M. Suess , M. R. Uehling , W. Kaminsky , G. Lalic , J. Am. Chem. Soc. 2015, 137, 7747–7753. 26042355 10.1021/jacs.5b03086

[25] S. Zhang , H. Fallah , E. J. Gardner , S. Kundu , J. A. Bertke , T. R. Cundari , T. H. Warren , Angew. Chem. Int. Ed. 2016, 55, 9927–9931; 10.1002/anie.201603970 27409068

[26] C. M. Zall , J. C. Linehan , A. M. Appel , J. Am. Chem. Soc. 2016, 138, 9968–9977. 27434540 10.1021/jacs.6b05349

[27] A. L. Speelman , B. L. Tran , J. D. Erickson , M. Vasiliu , D. A. Dixon , R. M. Bullock , Chem. Sci. 2021, 12, 11495–11505. 34567502 10.1039/d1sc01911b PMC8409461

[28] A. J. Jordan , C. M. Wyss , J. Bacsa , J. P. Sadighi , Organometallics 2016, 35, 613–616.

[29] L. Zhang , J. Cheng , Z. Hou , Chem. Commun. 2013, 49, 4782–4784. 10.1039/c3cc41838c 23598425

[30] B. L. Tran , B. D. Neisen , A. L. Speelman , T. Gunasekara , E. S. Wiedner , R. M. Bullock , Angew. Chem. Int. Ed. 2020, 59, 8645–8653; 10.1002/anie.201916406 32022415

[31] C. Kleeberg , Z. Anorg. Allg. Chem. 2011, 637, 1790–1794.

[32] Deposition Number 2145253 contains the supplementary crystallographic data for this paper. These data are provided free of charge by the joint Cambridge Crystallographic Data Centre and Fachinformationszentrum Karlsruhe Access Structures service.

[33] M. Brookhart , B. Grant , A. F. Volpe , Organometallics 1992, 11, 3920–3922.

[34] B. H. Lipshutz , W. Chrisman , K. Noson , J. Organomet. Chem. 2001, 624, 367–371.

[35] O. G. Shirobokov , L. G. Kuzmina , G. I. Nikonov , J. Am. Chem. Soc. 2011, 133, 6487–6489. 21476545 10.1021/ja111748u

[36] T. Gathy , D. Peeters , T. Leyssens , J. Organomet. Chem. 2009, 694, 3943–3950.

[37] T. Vergote , F. Nahra , A. Merschaert , O. Riant , D. Peeters , T. Leyssens , Organometallics 2014, 33, 1953–1963.

