# Supplementary material for: A Well‐Defined Anionic Dicopper(I) Monohydride Complex that Reacts like a Cluster
Source: Angew Chem Int Ed Engl. 2022 May 9;61(29):e202202318. doi: 10.1002/anie.202202318 (PMC9400846; doi:10.1002/anie.202202318)
Supplement: Supplementary file 1 — Supporting Information [file ANIE-61-0-s001.pdf]

Angewandte Chemie
Eine Zeitschrift der Gesellschaft Deutscher Chemiker

Supporting Information

A Well-Defined Anionic Dicopper(I) Monohydride Complex that Reacts like a Cluster

*R. L. M. Bienenmann, A. J. Schanz, P. L. Ooms, M. Lutz, D. L. J. Broere**

Content

Dinuclear copper hydrides in literature ... 3
Spacefill model of complex A ... 5
Experimental methods ... 6
General considerations ... 6
Synthesis of complex **1** ... 6
Synthesis of complex **1D** *in situ* ... 10
Exposure of complex **1** to air ... 10
Reaction of **1** with D_2SiPh_2 ... 12
Protonation of **1** with $HBArF_{24}$•$2Et_2O$ ... 13
Synthesis of $HSiPh_2(OCHPH_2)$ ... 15
Reaction of **1** with 4-fluorophenyl acetylene ... 16
Catalysis ... 20
Representative procedure for screening unsaturated substrates for hydrosilylation with triphenyl silane catalyzed by **1** ... 20
Hydrosilylation of benzophenone with triphenylsilane catalyzed by complex **1** ... 20
Hydrosilylation of 4-chloro benzaldehyde with triphenylsilane catalyzed by **1** ... 21
Hydrosilylation of benzophenone with diphenylsilane catalyzed by **1** ... 23
Stoichiometric hydrosilylation of benzophenone with diphenylsilane-d_2 catalyzed by **1** ... 23
Silane scrambling of (diphenylmethoxy)diphenylsilane catalyzed by **1** ... 24
Hydrosilylation of benzophenone with triphenylsilane catalyzed by [PNNP**Cu2Mes]K(18-crown-6) ... 25
Hydrosilylation of benzophenone with triphenylsilane using Stryker's reagent as catalyst ... 26
Computational methods ... 28
General remarks ... 28
Geometry optimizations ... 28
Bonding analysis ... 31
Hydride charge comparison ... 32
Tracking charge upon deprotonation ... 32
X-ray crystal structure determination ... 34
References ... 35

Dinuclear copper hydrides in literature

Table S1: An overview of literature reported dicopper hydride complexes. Dipp = 2,5-diisopropylphenyl, R^1=di(4-tert-butylphenyl)methyl, R^2 = diphenylmethyl, R^3 = Cl, Me, Et, OMe or tBu.

Structure	***Reference***
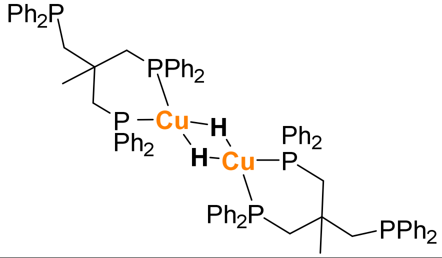	Goeden et al. 1986[1]
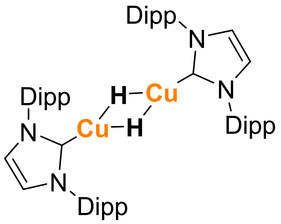	Mankad et al. 2004[2]
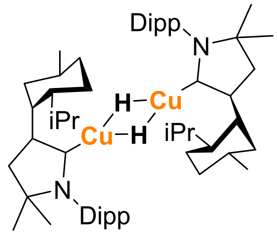	Frey et al. 2011[3]
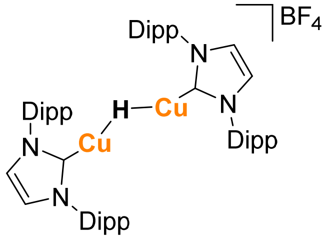	Wyss et al. 2013[4]
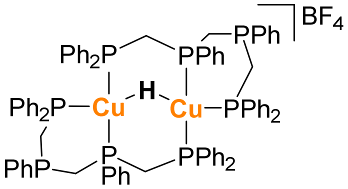	Nakamae et al. 2014[5]
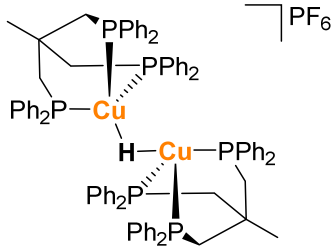	Zall et al. 2016[6]

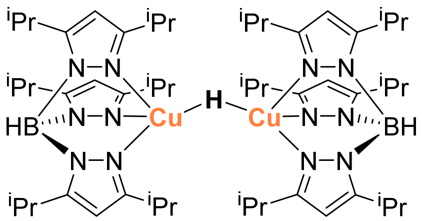	Zhang et al. 2016[7]
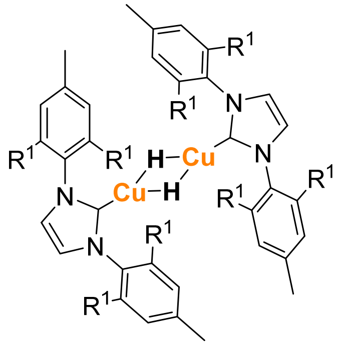	Romero et al. 2017[8]
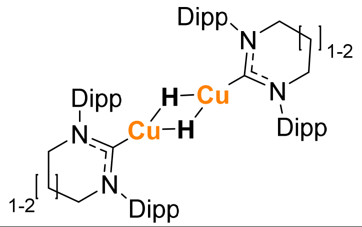	Jordan et al. 2016[9]<br>Tran et al. 2020 (crystal structure)[10]
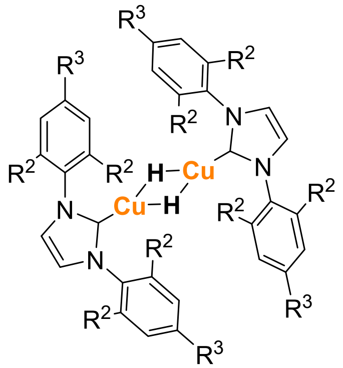	Speelman et al. 2021[11]

These structures were found with a Conquest search of the CCDC database.[12] A combination of two queries was used. The first is of the type “drawn”, where two copper atoms bridged by a hydrogen atom that is connected to each Cu atom with bond type “any” were searched. The second query is of type “formula” and searches for Cu_2 (excluding any structure with 1 or more than 2 copper atoms). Structures with transition metals other than copper in the Cu_2H core were omitted as well as structures with bridging borohydrides. This search did not yield the 2004 structure of Sadighi and coworkers[2] due to the absence of the hydride ligands in the crystallographic data.

Spacefill model of complex A

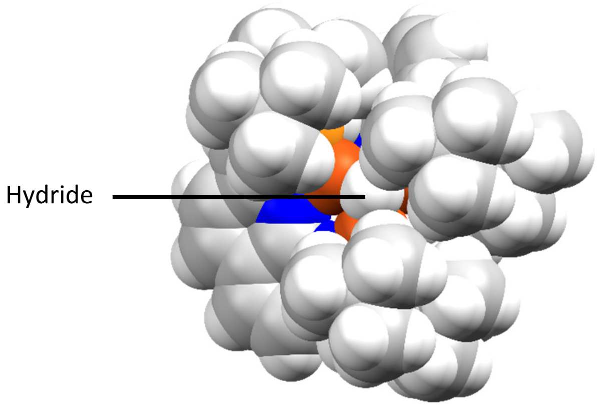

*Figure S1: Spacefill model of complex **A** generated from the reported crystal structure.*[13]

Experimental methods

General considerations

All manipulations were performed under N_2 atmosphere in a glovebox or using Schlenk techniques, with anhydrous reagents unless mentioned otherwise. Ambient temperature in our glovebox is on average 27°C. Glassware was oven dried at 130°C overnight or flame dried before use. Solvents except THF were collected from an SPS system and degassed by bubbling N_2 gas through it, subsequently they were dried over molecular sieves. The THF was distilled over Na/benzophenone ketyl (purple), subsequently it was degassed by bubbling N_2 gas and further dried over molecular sieves. Water content in the solvents was tested using Karl-Fisher titration and titration with a ketyl solution. Deuterated solvents were degassed using three freeze pump thaw cycles and dried over molecular sieves. The PNNP ligand as well as complex **A** and [$^{t\text{-}Bu}$PNNP**Cu_2Mes]K18-crown-6 were prepared according to literature procedure.[13,14] $HBArF_{24}$•$2Et_2O$ (Brookhart's acid) was prepared according to literature procedure.[15] All commercial reagents were obtained from Sigma Aldrich or Acros, liquids were degassed using three freeze pump thaw cycles and dried over molecular sieves before use. 4-chlorobenzaldehyde was sublimed to eliminate the benzoic acid oxidation product and subsequently stored in the glovebox. NMR spectra were recorded on an Varian MRF 400 equipped with a OneNMR probe and Optima Tune system or a Varian VNMR-S-400 equipped with a PFG probe. All resonances in ^{1}H NMR were referenced to residual solvent peaks.[16] IR-data was recorded on a PerkinElmer SpectrumTwo Infrared Spectrophotometer equipped with an ATR-probe. Elemental analysis was performed by MEDAC ltd. in the United Kingdom.

Synthesis of complex 1

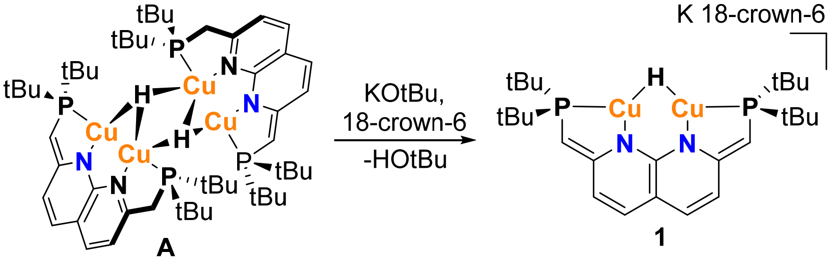

A solution of KO^{t}Bu (11.4 mg, 94.1 µmol, 2.3 eq) and 18-C-6 (24.7 mg, 93.4 µmol, 2.3 eq) in THF (2 mL) was added dropwise to a stirred red solution of **A** (47.5 mg, 41.4 µmol) in THF (5 mL) at ambient temperature. The reaction mixture gradually turned orange and was left to stir for one hour. Subsequently, the solvent was evaporated and the residual orange solid was washed with *n*-pentane (7 ml). The residue was extracted with THF until washings were colorless (~7 mL), extracts were combined and all volatiles were evaporated under a dynamic vacuum to give **1** as an orange solid in 75% yield (63.3 mg, 61.9 µmol). Complex **1** can be stored as a solid at -40°C under N_2 atmosphere for 2-3 weeks without a visual change in color and morphology. The NMR spectra of **1** contain a minor impurity of **A**, likely formed during sample preparation due to the high sensitivity of **1**.

Single crystals of **1** suitable for X-ray diffraction were grown by vapor diffusion of pentane into a concentrated THF solution of the compound.

^{1}H NMR (400 MHz, THF-*d*$_8$, 298 K): δ 6.12 (d, $^3J_{H,H}$ = 8.3 Hz, 2H), 5.55 (d, $^3J_{H,H}$ = 8.3 Hz, 2H), 3.22 (s, 2H), 1.17 (d, $^2J_{H,P}$ = 12.0 Hz, 36H), 0.89 (t, $^3J_{P,H}$ = 25.3 Hz, 1H) ppm.

^{31}P{^{1}H} NMR (162 MHz, THF-*d*$_8$, 298 K): δ 20.5 (s) ppm.

13 C {¹H} APT (101 MHz, THF-d_8, 298 K): δ 169.4 (d, *J* = 19.3Hz), 156.8, 130.7, 108.8 (m due to virtual coupling), 103.7, 71.0 (18-C-6), 62.5 (d, $^1J_{C,P}$ = 46.5 Hz), 33.5 (m due to virtual coupling), 30.5 (m due to virtual coupling) ppm.

IR-ATR (cm^{-1}): 2890 (s), 1618 (m), 1579 (m), 1538 (m), 1499 (s), 1469 (w), 1419 (s), 1352 (m), 1322 (w), 1249 (w), 1179 (w), 1111 (s), 962 (m), 822 (m), 789 (m), 588 (w).

Anal. Calc. for $C_{46}H_{83}Cu_2KN_2O_8P_2$: C, 54.15; H, 8.20; N, 2.74. Found C, 53.60; H, 8.43; N, 2.75. *

*The extremely reactive nature of **1** precluded a satisfactory elemental analysis. Given the fact that under the extremely rigorous dry conditions that our group works we still often see partial protonation of **1**, we deem it likely that a commercial company performing elemental analysis cannot prevent picking up some trace moisture during sample preparation. For example, the inclusion of 0.5 eq of H_2O gives a calculated CHN ratio (C, 53.68; H, 8.23; N, 2.72), that is in agreement with the found values.

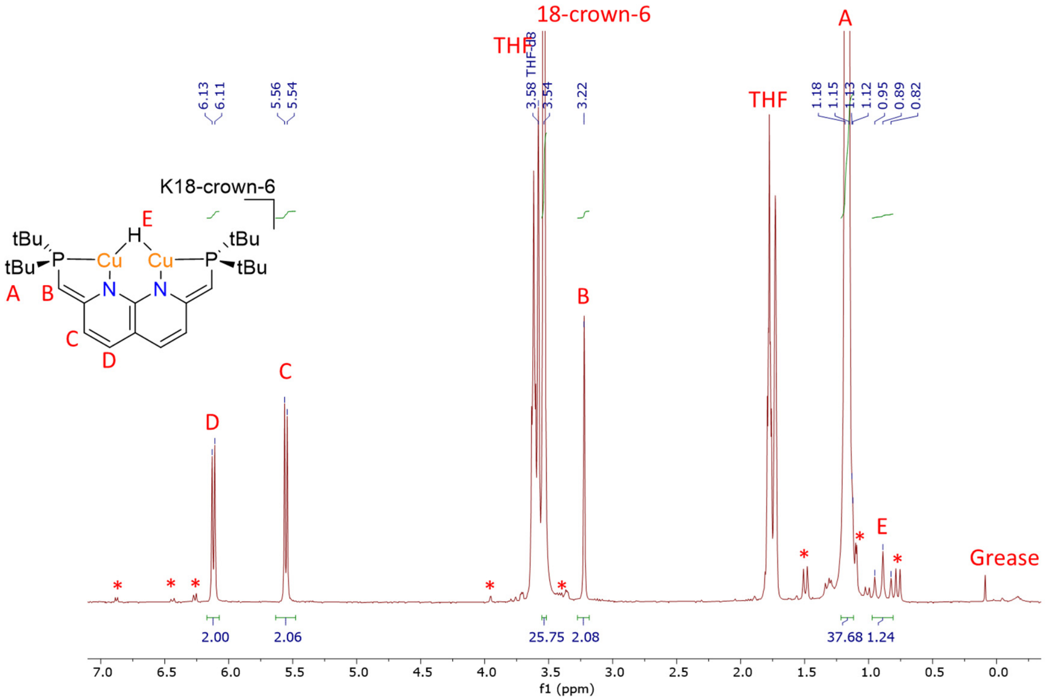

*Figure S2: ¹H NMR spectrum of **1** in THF-d_8 at 25°C, * marks peaks belonging to **A**.*

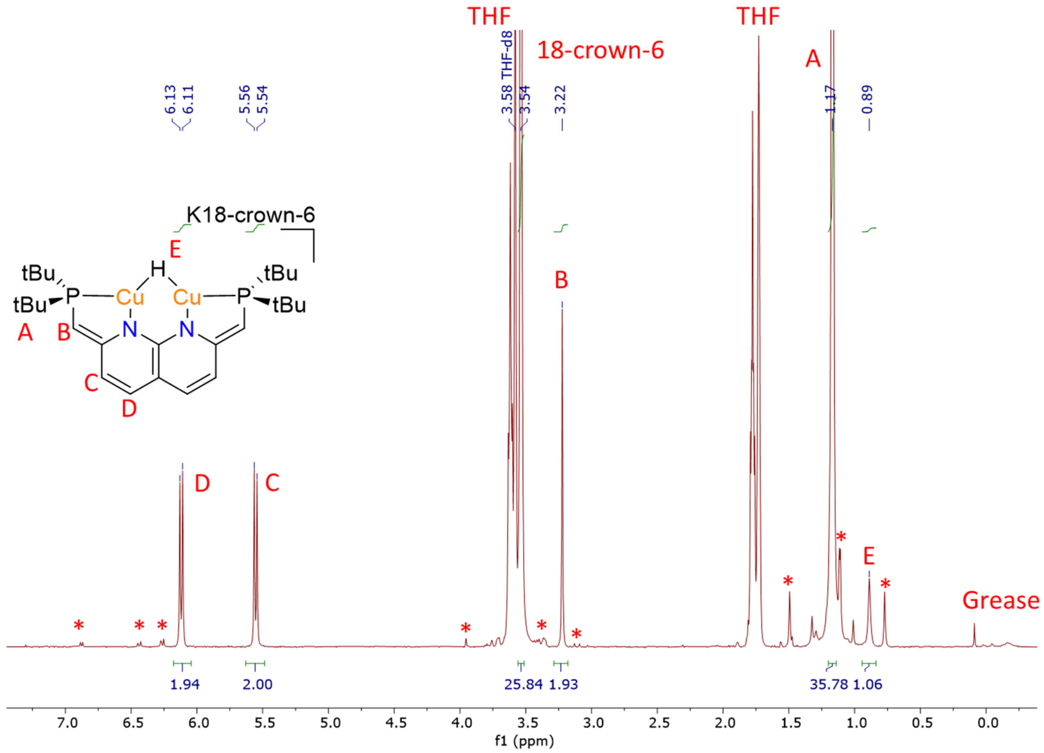

*Figure S3: $^{1}H\{^{31}P\}$ NMR spectrum of **1** in THF-d_8 at 25°C, * marks peaks belonging to **A**.*

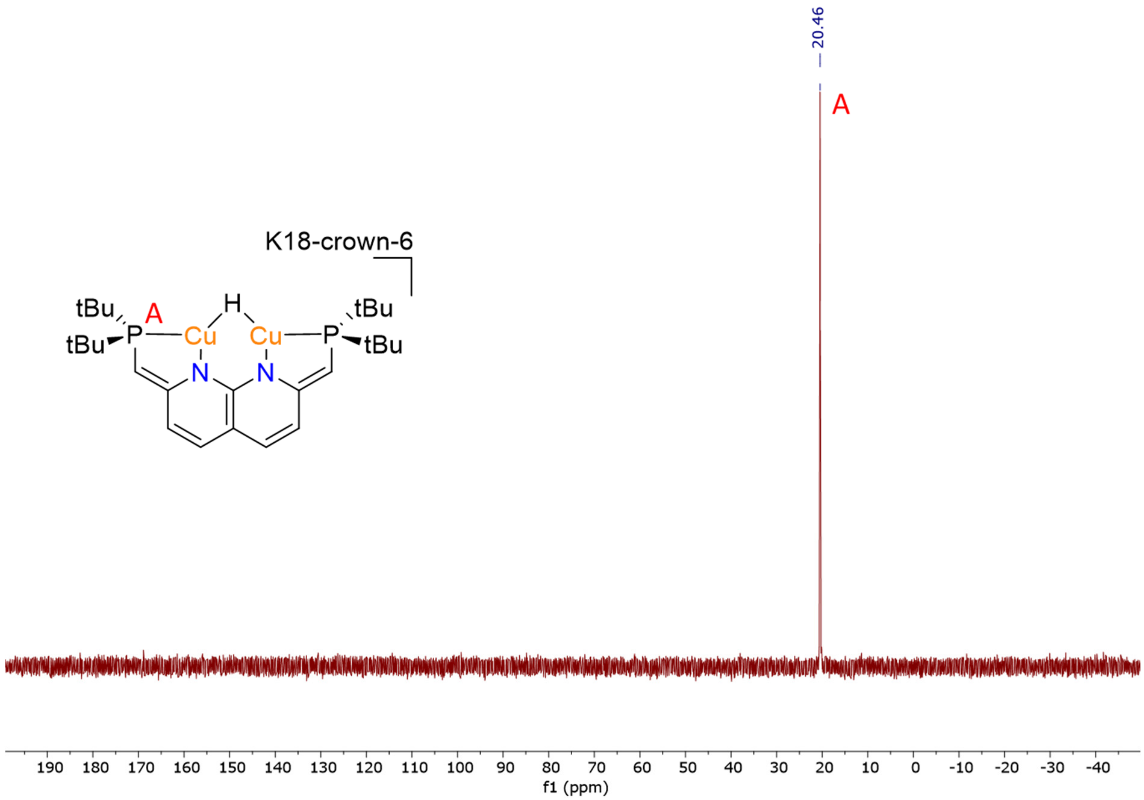

*Figure S4: ^{31}P NMR spectrum of **1** in THF-d_8 at 25°C.*

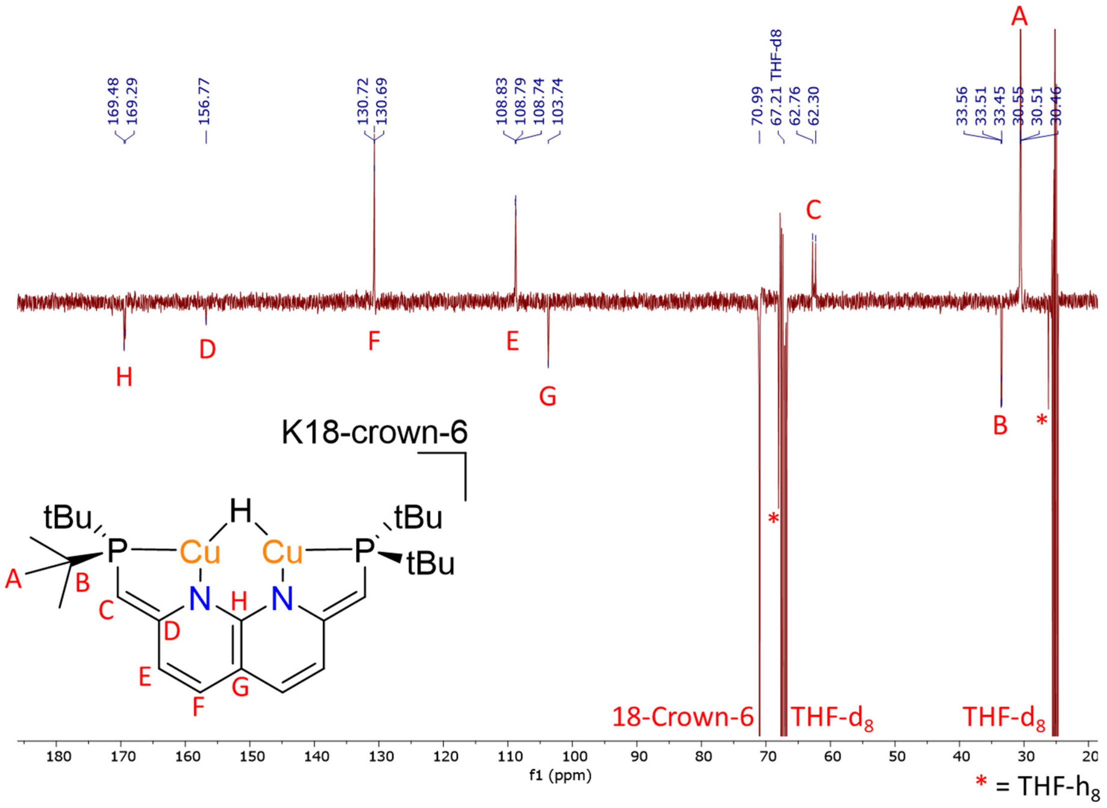

*Figure S5: ^{13}C (APT) NMR spectrum of **1** in THF-d_8 at 25°C.*

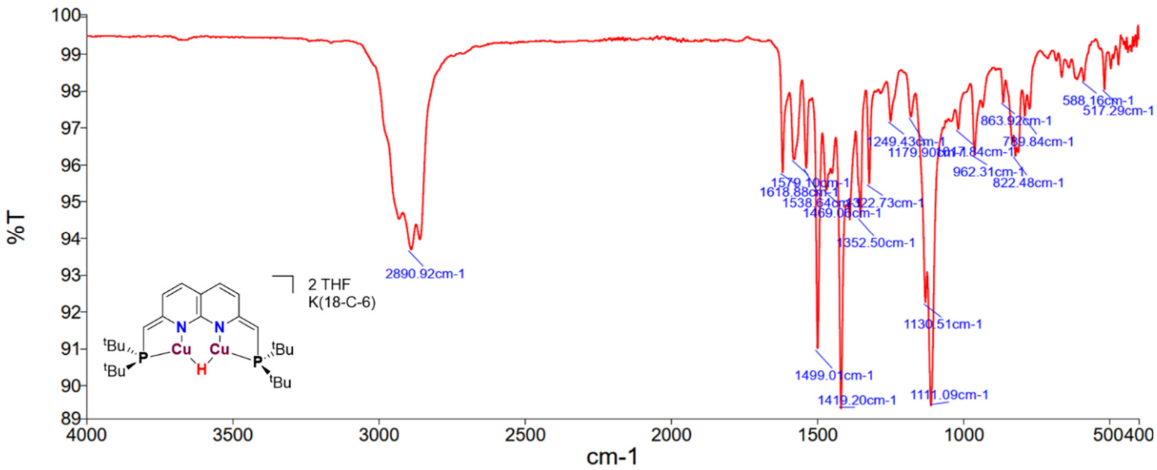

*Figure S6: IR Spectrum of **1**.*

Synthesis of complex 1D *in situ*

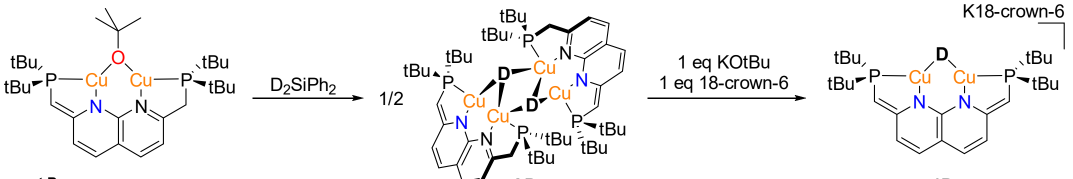

t-BuPNNP*Cu2OtBu — AD — 1D

The deuterated analogue of **A** (**AD**) was prepared according to literature procedure.[13] Without isolating **AD**, 18-crown-6 (19.2 mg, 72.6 µmol, 2.4 eq) and KOtBu (7.8 mg, 69.5 µmol, 2.3 eq) in 0.2 ml THF were added dropwise to a THF (0.5ml) solution of **AD** (1eq, 30.9 µmol). The mixture was left to stir for 20 mins and was subsequently analyzed by ^{2}H NMR spectroscopy.

^{2}H NMR (61 MHz, THF-h$_8$): δ 0.95 (s) ppm.

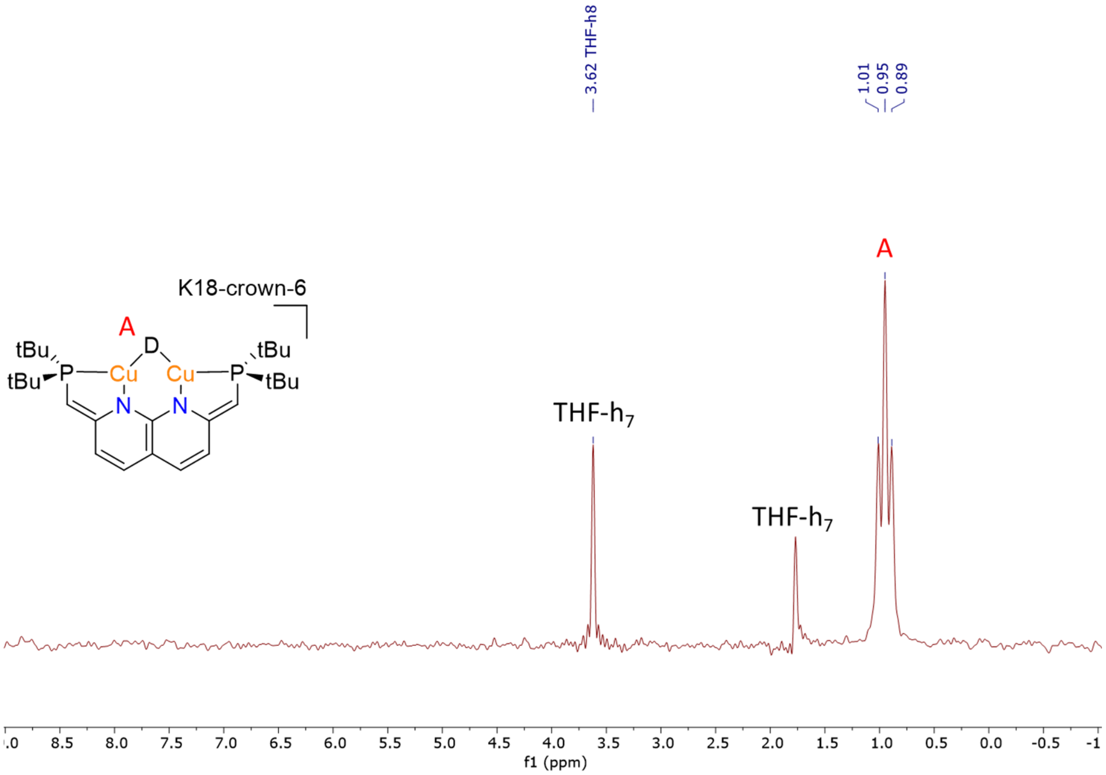

*Figure S7: ^{2}H NMR spectrum of **1D** in THF-h$_8$ at 25°C.*

Exposure of complex 1 to air

Complex **1** (5.6 mg, 5.5 µmol) was dissolved in THF (0.6 ml) and sealed in a J-Young tube. On the Schlenk line, the solution was degassed with three freeze-pump-thaw cycles. The tube was backfilled with air by opening the tube and closing it again. NMR of the solution shows mostly the peaks reported for **A** along with some smaller unidentified species which are hypothesized to be oxidation products.

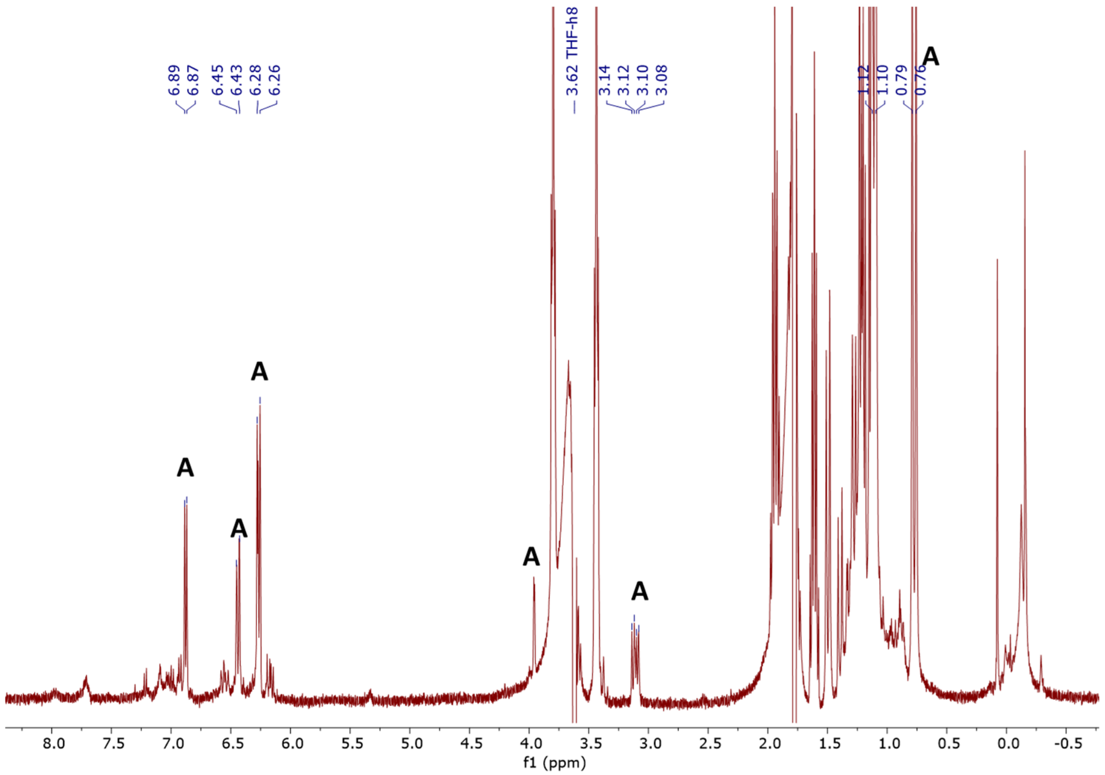

*Figure S8: ^{1}H NMR spectrum of **1** after exposure to air in THF-h_8 at 25°C with PRESAT solvent suppression for the THF peaks.*

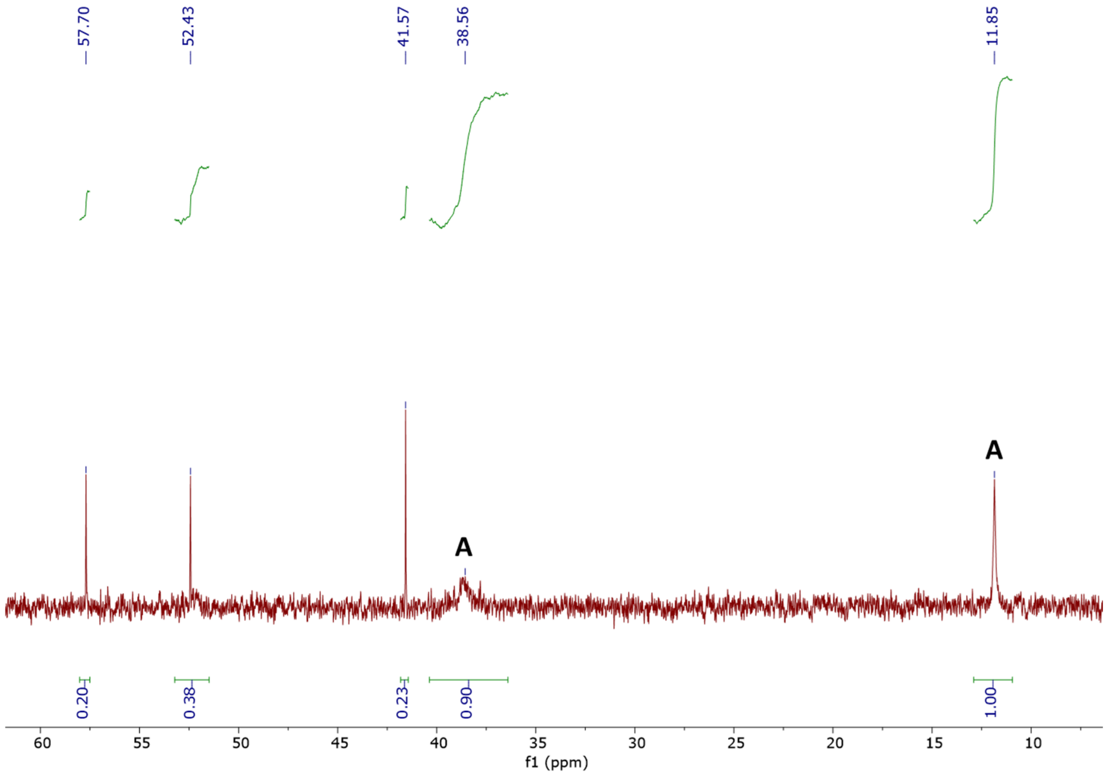

*Figure S9: ^{31}P NMR spectrum of **1** after exposure to air in THF-h_8 at 25°C.*

Reaction of **1** with D_2SiPh_2

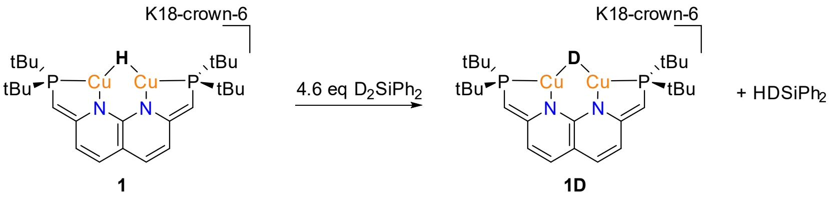

Complex **1** (1 eq, 4.8 mg, 4.7 µmol) was dissolved in 0.65 ml THF and transferred to a J-Young tube. D_2SiPh_2 (4.6 eq, 4 µl, 21.6 µmol) was added to the tube and the solution was mixed by shaking. After 1.5 hours the mixture was analyzed by ^1H and ^2H NMR spectroscopy, which showed the formation of **1D** concomitantly with the formation of $HDSiPh_2$.

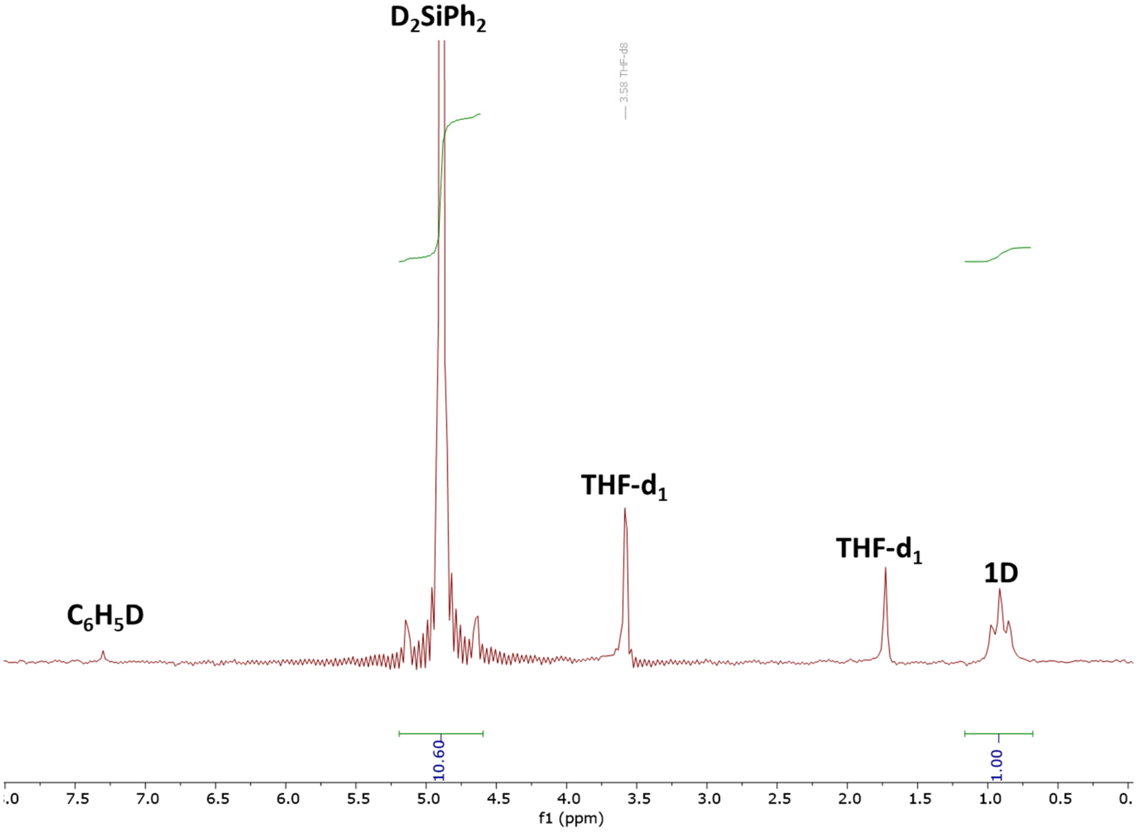

*Figure S10: ^2H NMR (THF-h_8, 298K) spectrum of the reaction of **1** with D_2SiPh_2.*

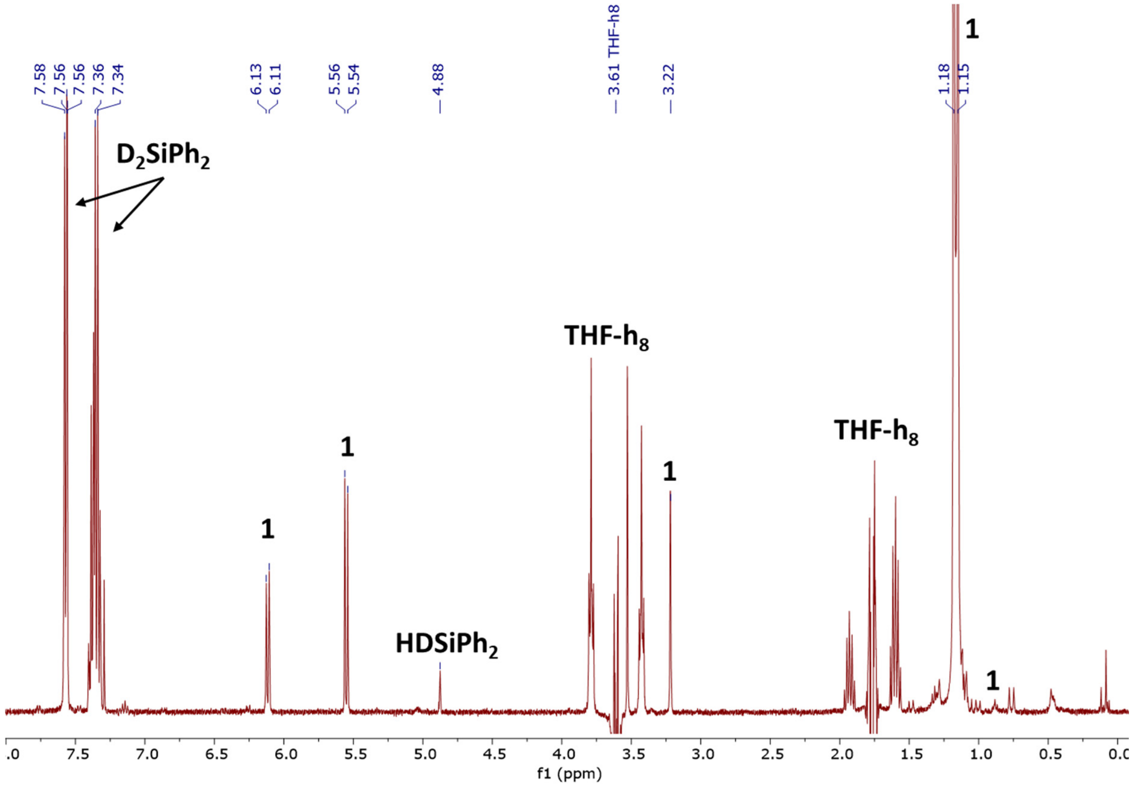

*Figure S11: ^{1}H NMR spectrum (THF-h_8, 298K) of the reaction of **1** with D_2SiPh_2 with solvent suppression (PRESAT) for the THF solvent peaks.*

Protonation of 1 with $HBArF_{24}$•$2Et_2O$

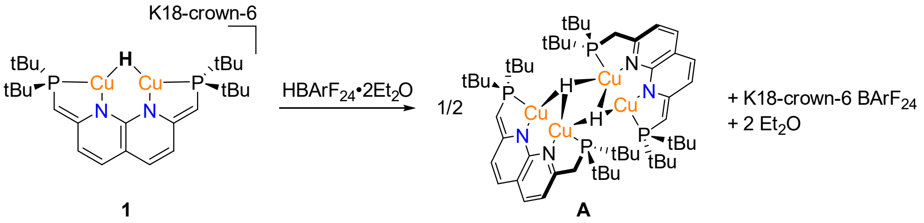

Complex **1** (1 eq, 20.0 mg, 19.6 µmol) was dissolved in THF (1.5 ml), and $HBArF_{24}$•$2Et_2O$ (1 eq, 21.2 mg, 19.6 µmol) in THF (1.5 ml) was added dropwise to the stirring solution of **1**. During the addition, the mixture turned from orange to red. Both ^{1}H NMR and ^{31}P NMR showed the formation of complex **A**, the NMR data of **A** matches those reported in literature.[13]

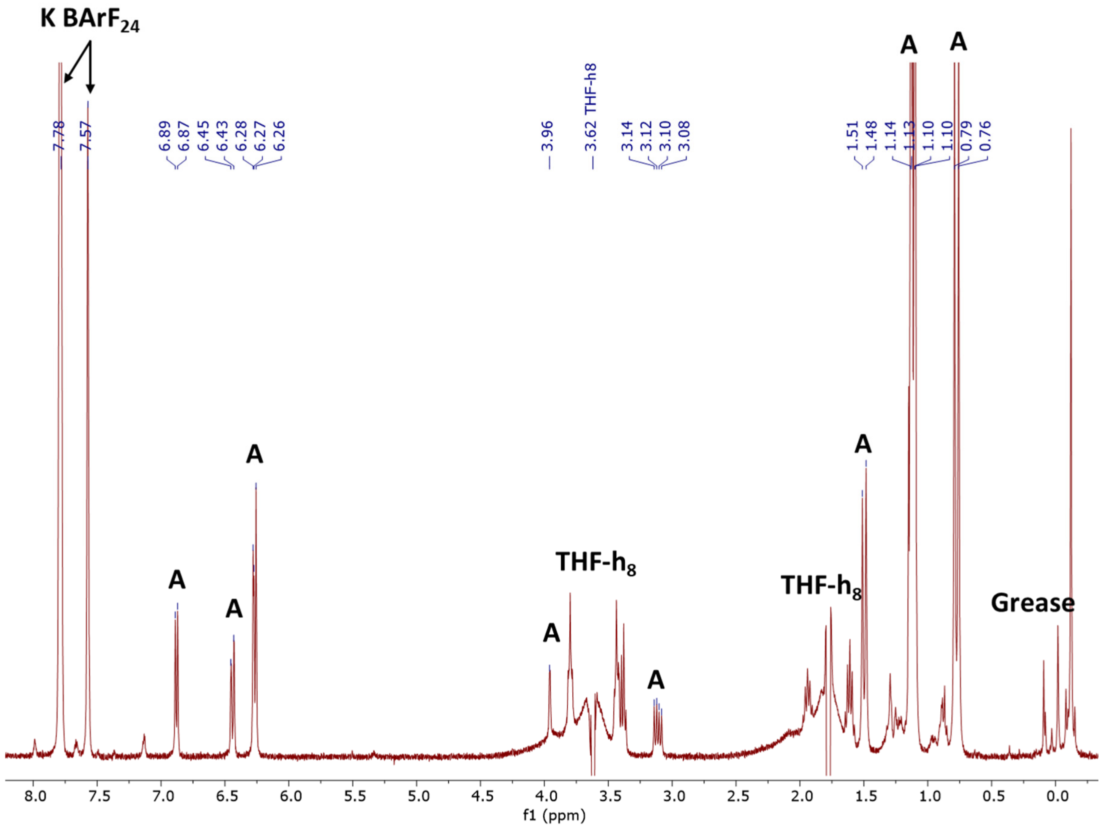

*Figure S12: ^{1}H NMR spectrum of the product of **1** with 1eq HBArF$_{24}$ in THF-h$_8$ at 25°C with PRESAT solvent suppression for the THF peaks.*

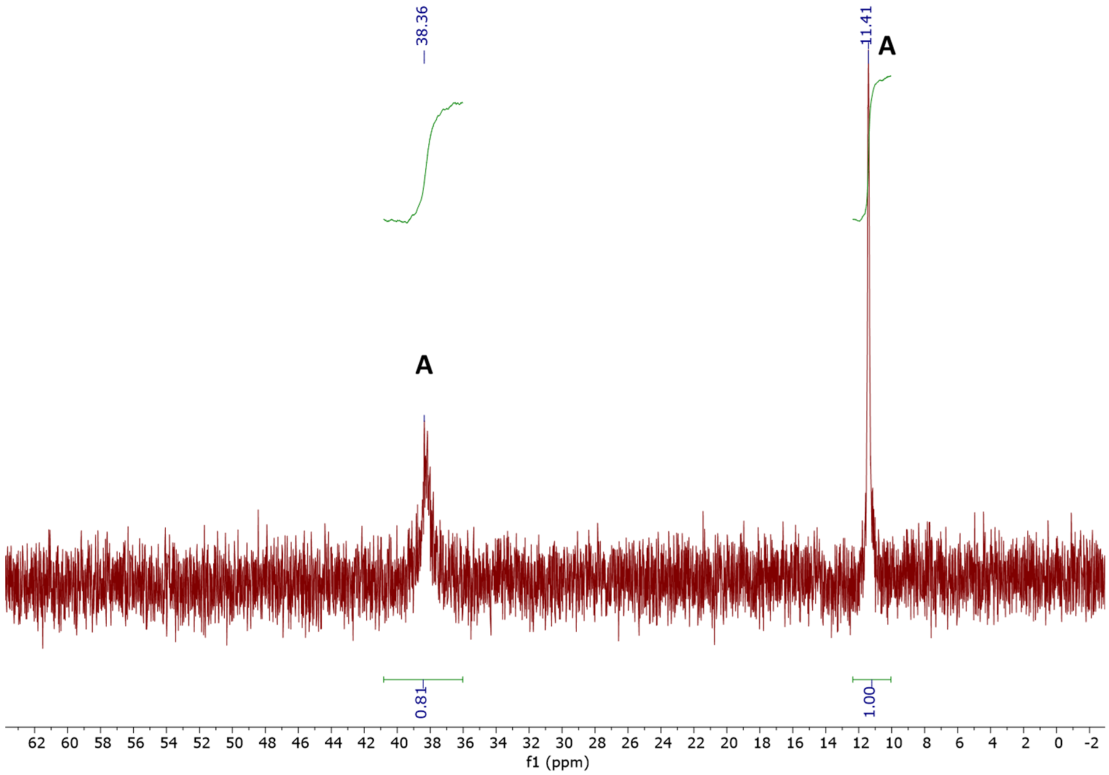

*Figure S13: ^{31}P NMR spectrum of **1** with 1eq HBArF$_{24}$ in THF-h$_8$ at 25°C.*

Synthesis of $HSiPh_2(OCHPH_2)$

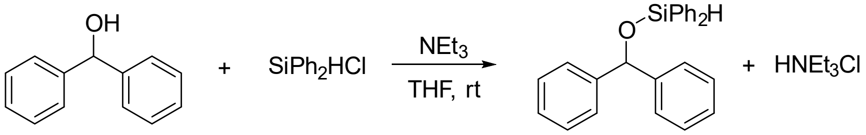

According to modified literature procedure[17], benzhydrol (204.99 mg, 1.11 mmol, 1 eq) was dissolved in THF (15 ml). Diphenylchlorosilane (0.22 ml, 1.13 mmol, 1 eq) was added dropwise to the stirring solution of benzhydrol. The mixture was stirred for 3 hours after which NMR showed only minimal conversion. Triethylamine (0.15 ml, 1.08 mmol, 0.95 eq) was added dropwise to the stirring solution, which led to the immediate formation of a white precipitate. The solvent was evaporated and the white solid was extracted with hexane (6 ml). The extract was dried and recrystallized from 3.3 ml hexane at -40 °C yielding a white powder (48%, 195.9 mg, 0.53 mmol). NMR analysis is consistent with the reported values.[18]

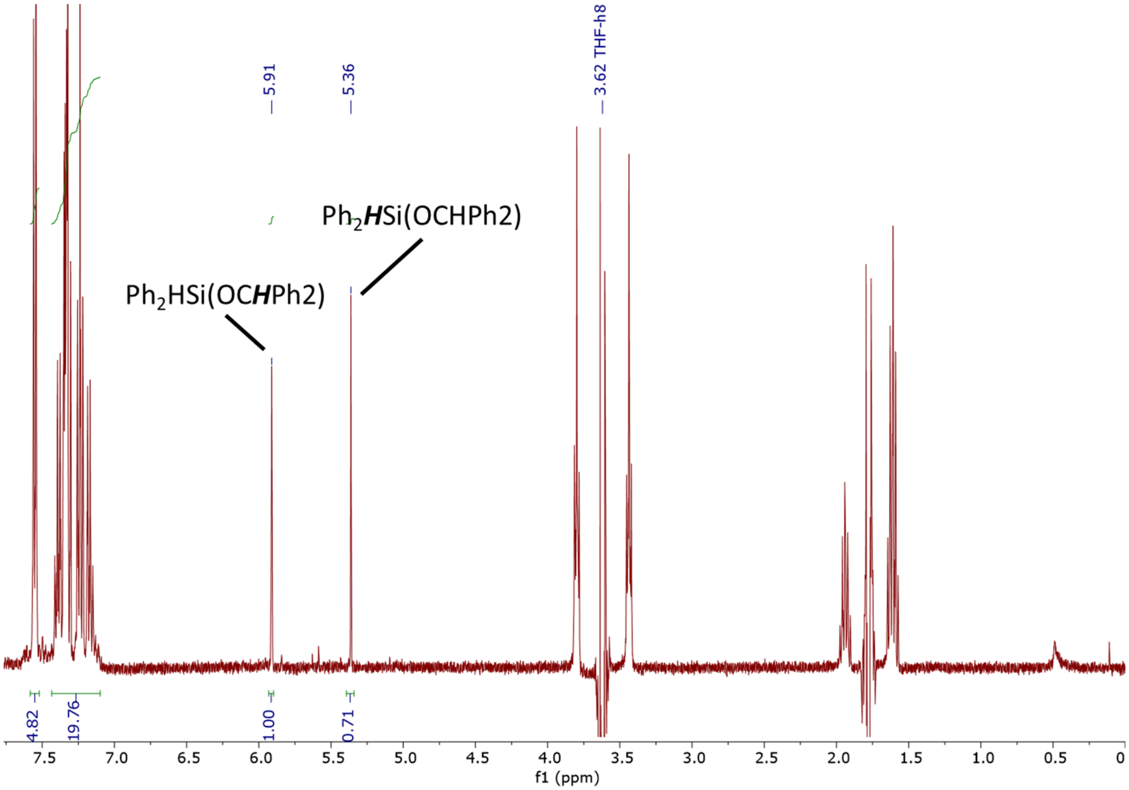

Figure S14: ^1H NMR spectrum of $Ph_2HSi(OCHPh_2)$ in THF-h_8 at 25°C with PRESAT solvent suppression for the THF peaks.

Reaction of 1 with 4-fluorophenyl acetylene

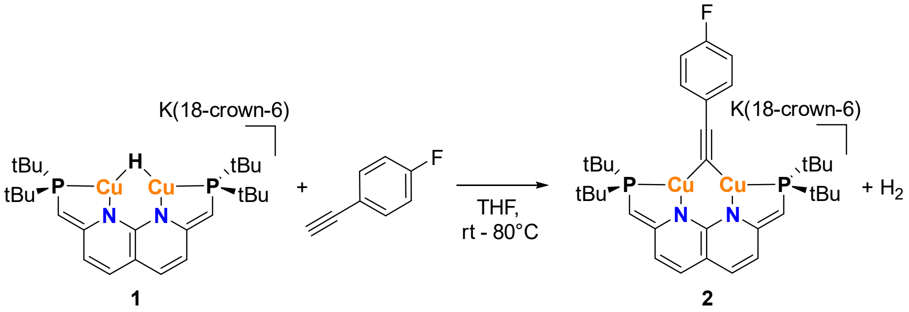

Complex **1** (15.1 mg, 14.7 µmol) was dissolved in THF-d_8 (0.6 ml) and transferred to a J-Young tube. 4-fluoro phenylacetylene (1.7 µL, 14.7 µmol, 1 eq) was added. The tube was closed. The sample was heated stepwise and monitored with NMR. First at 40°C overnight, then 3 hours at 50°C, then 60°C overnight, then 70°C for 3 hours and finally 80°C overnight.

^{1}H NMR (400 MHz, THF-d_8, 298 K): δ 7.59 – 7.40 (m, 2H, *side-product*), 7.25 – 7.16 (m, 2H), 7.11 – 7.00 (m, 2H, *side-product*), 6.87 – 6.75 (m, 2H), 6.11 (d, $^{3}J_{H,H}$ = 8.5 Hz, 2H), 5.53 (d, $^{3}J_{H,H}$ = 8.4 Hz, 2H), 4.54 (s, H_2), 3.24 (d, $^{2}J_{H,P}$ = 2.4 Hz, 2H), 1.19 (d, $^{3}J_{H,P}$ = 12.3 Hz, 36H) ppm.

^{19}F{^{13}C} NMR (376 MHz ,THF-d_8, 298 K): δ -120.38 (m), *-116.04 (m, side-product)* ppm.

^{31}P{^{1}H} NMR (162 MHz, THF-d_8, 298 K): δ 9.6 (s) ppm.

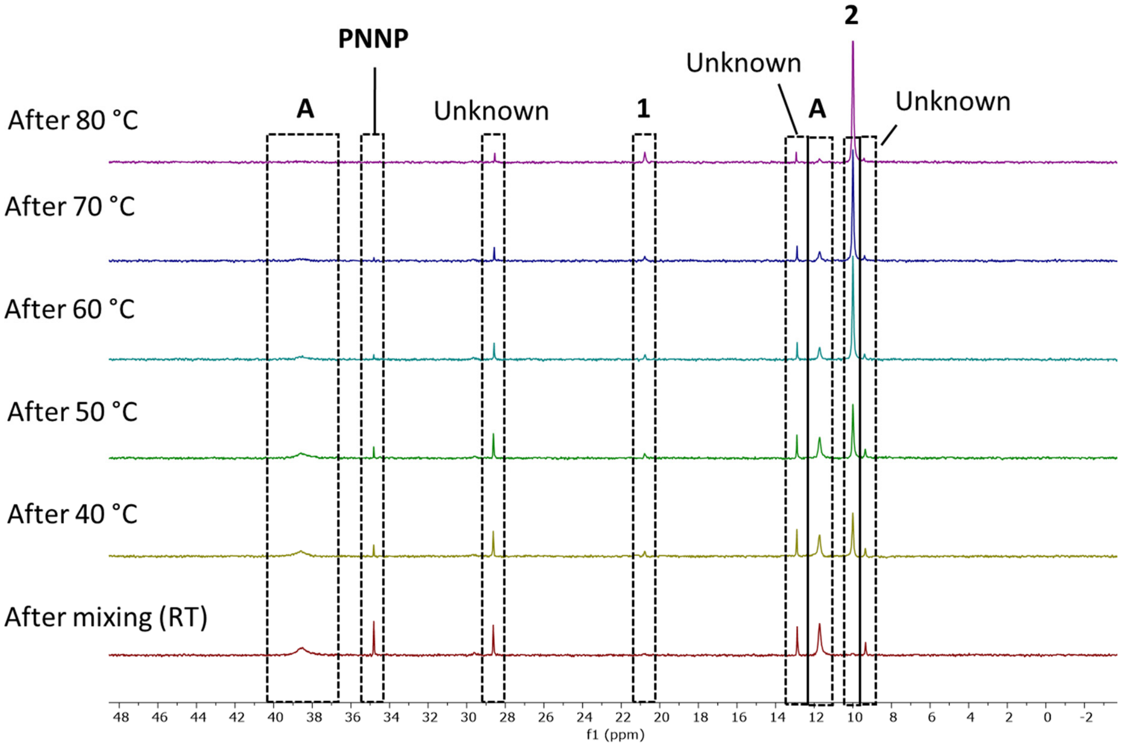

*Figure S15: ^{31}P NMR spectra of the reaction of **1** with 1 eq 4-fluorophenylacetylene in THF-d_8 at 25°C, monitored over time with incremental heating.*

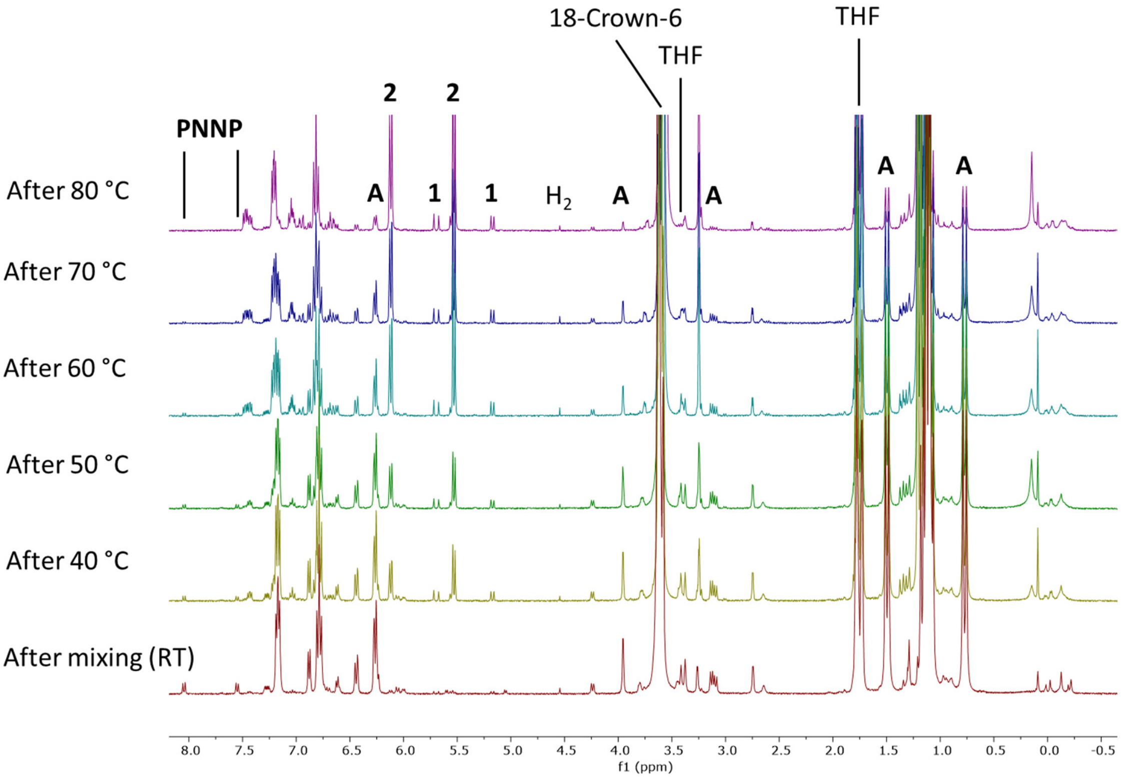

*Figure S16: ^{1}H NMR spectra of the reaction of **1** with 1 eq 4-fluorophenylacetylene in THF-d_8 at 25°C, monitored over time with incremental heating. Isolated peaks are assigned.*

Reaction of 1 with CO_2

Complex **1** (5.2 mg,) was dissolved in 0.6 ml THF and transferred to a J-Young tube. The tube was attached to a gas addition setup and degassed with three freeze-pump-thaw cycles. The tube was filled with CO_2 at room temperature. The mixture was analysed with NMR after 1 hour, 1 day and 2 days. After two days, a colourless precipitate had formed which was filtered off, washed with THF extensively until no orange colour was visible, and dissolved in D_2O showing the presence of formate.

We hypothesize that initially, the CO_2 is attacked by the backbone leading to the resonances at 4.70 and 4.75 ppm (dashed circle) after 1 hour. Resonances at similar position have been reported for a related mononuclear PNP CO_2 complexes.[19–21] After this, conversion to A and an unidentified species that also contains a partially dearomatized ligand backbone is observed. Moreover formate (presumably KO_2CH) starts visually precipitating, which was confirmed by 1H NMR analysis of the precipitate (Figure S19). The composition of the solution after 2 days does not change further, even after heating analogous samples at 80 °C.

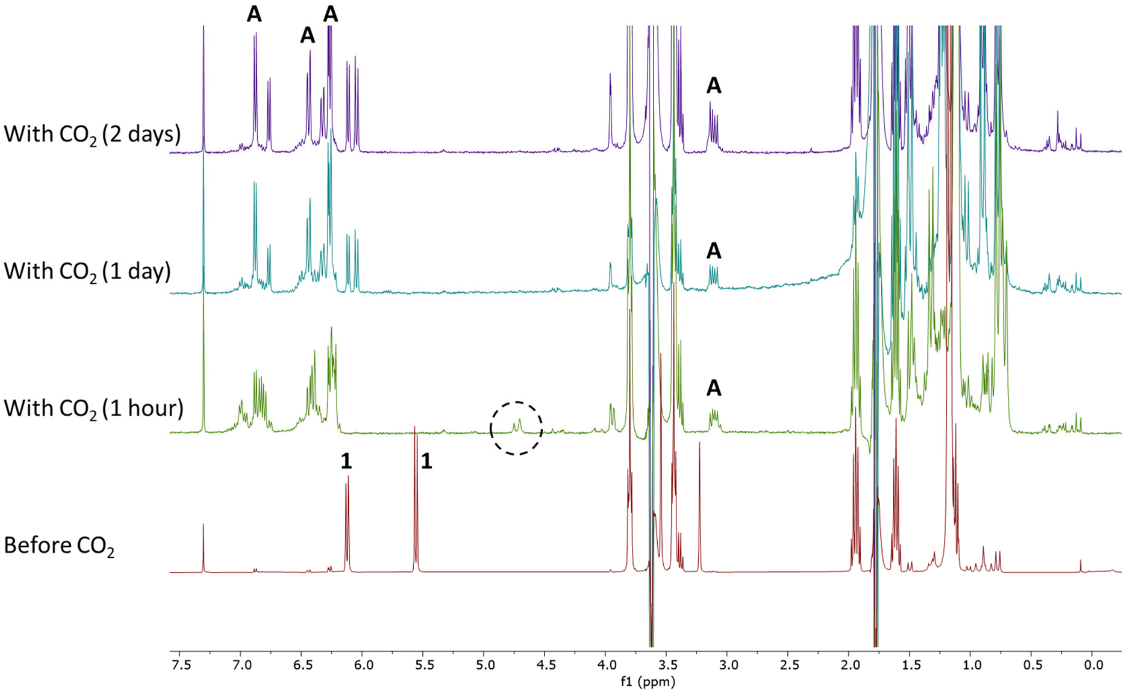

*Figure S17: ^{1}H NMR spectra of the reaction of **1** with CO_2 (1 atm.) in THF-h_8 at 25°C with presat solvent suppression for the THF peaks, monitored over time.*

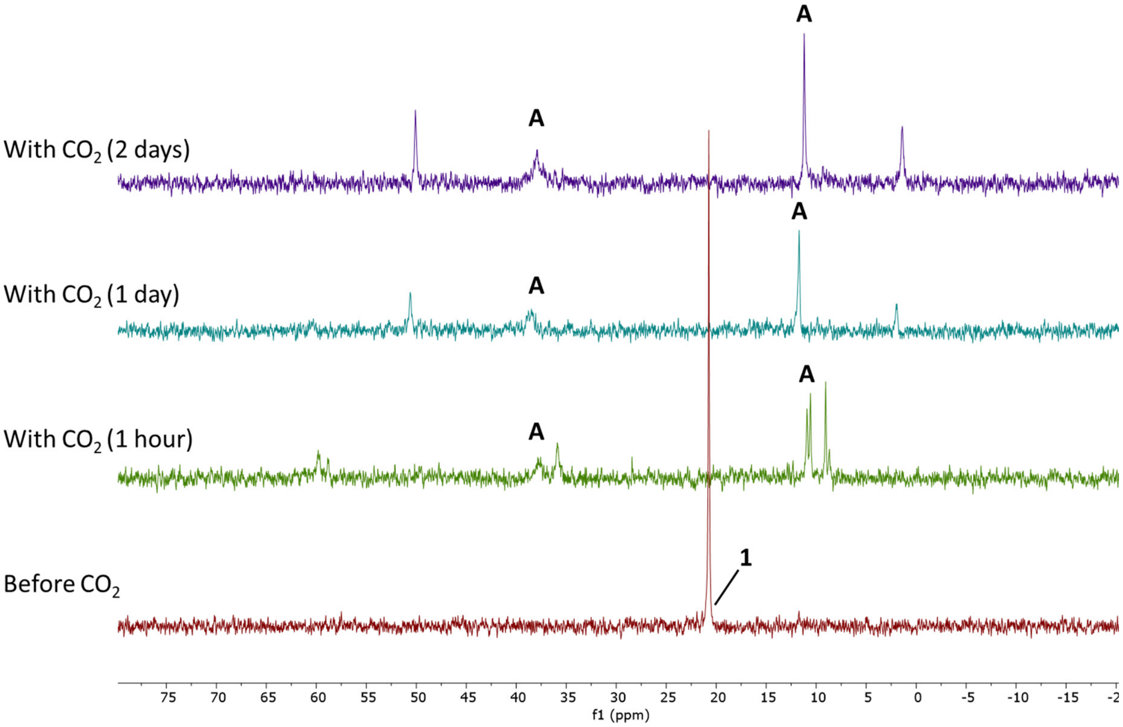

*Figure S18: ^{31}P NMR spectra of the reaction of **1** with CO_2 (1 atm.) in THF-d_8 at 25°C, monitored over time.*

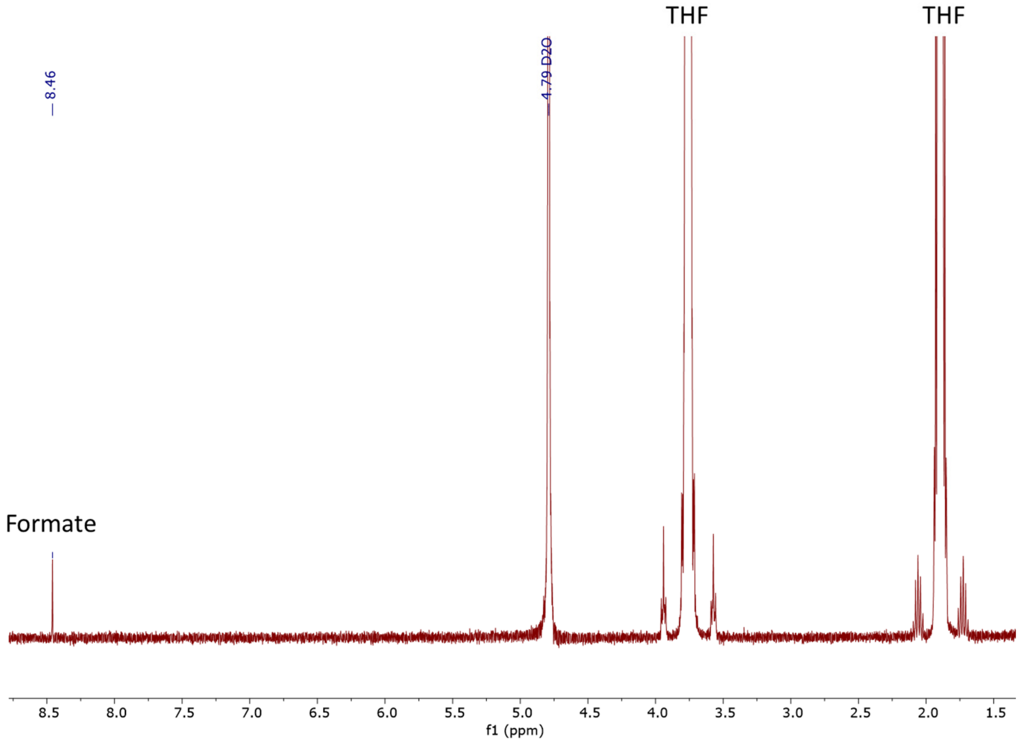

*Figure S19: ^{1}H NMR spectrum of the precipitate from reaction of **1** with CO_2 (1 atm.) in D_2O at 25°C.*

Catalysis

Representative procedure for screening unsaturated substrates for hydrosilylation with triphenyl silane catalyzed by 1

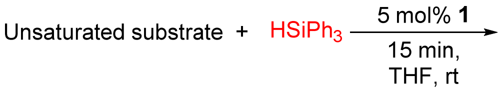

Triphenylsilane (1 eq, ~60 µmol) and complex **1** (5 mol%, ~3 µmol) were dissolved in THF (0.6 ml). Subsequently, the unsaturated substrate (1 eq, ~60 µmol) was added and after 15 minutes, the sample was analyzed with NMR. Benzonitrile, Butylacrylate, methylbenzoate and benzophenone imine were screened as substrates using this procedure.

Hydrosilylation of benzophenone with triphenylsilane catalyzed by complex 1

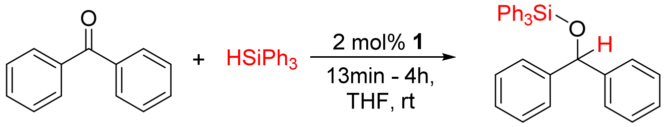

Complex **1** (2 mol%, 1.3 mg, 1.3 µmol) was dissolved in 0.5 ml of a THF solution containing mesitylene (0.4 eq, 25.2 µmol) and triphenylsilane (1 eq, 63.3 µmol). To this solution, benzophenone (1eq, 64.8 µmol) dissolved in THF (0.5 ml) was added. After 13 min,190 min and after 4 hours the sample was analyzed by NMR (4 scans, 20 seconds relaxation delay), monitoring the $Ph_3Si\underline{H}$ peak (5.41 ppm) the $Ph_2C\underline{H}OSiPh_3$ (5.94 ppm) and the aromatic mesitylene signal (6.70 ppm) as internal standard to determine the progression of the reaction. After 4 hours, >99% spectroscopic yield was observed.

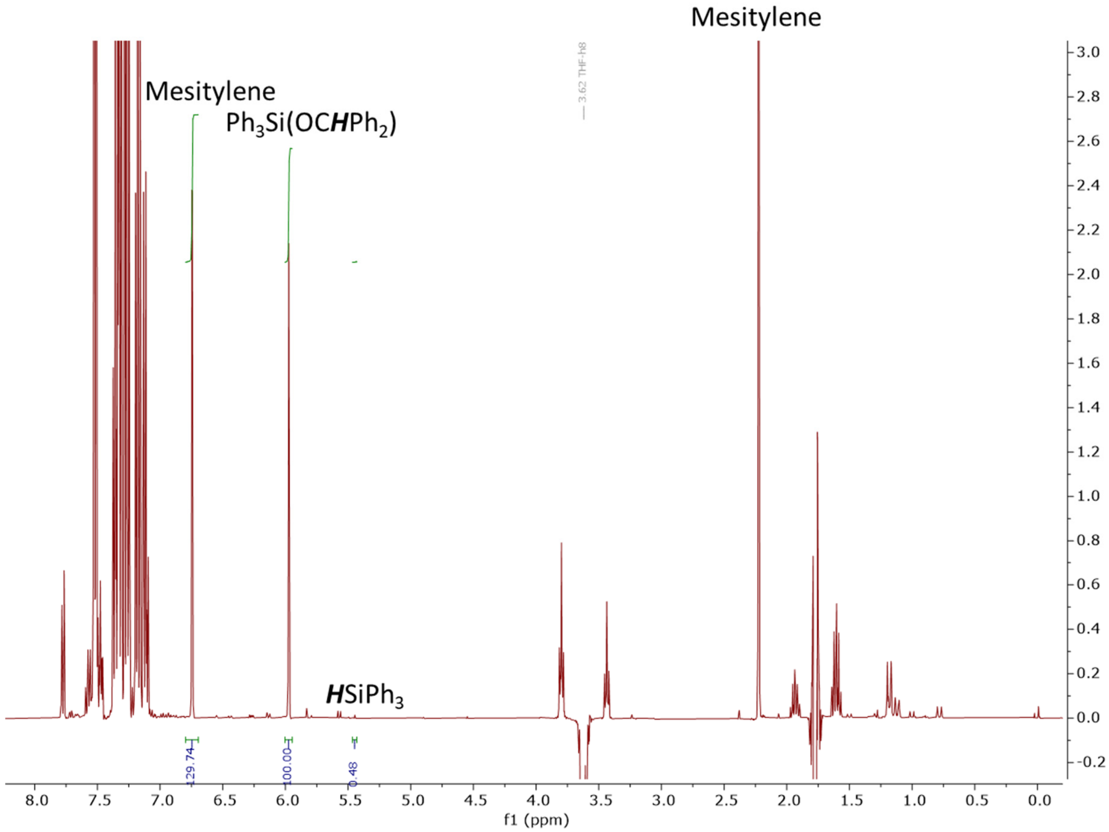

*Figure S20: ^{1}H NMR spectrum after 4 hours of the hydrosilylation of benzophenone with triphenylsilane with 2 mol% **1** as catalyst in THF-h_8 at 25°C with PRESAT solvent suppression for the THF peaks and 20 seconds relaxation delay.*

Hydrosilylation of 4-chloro benzaldehyde with triphenylsilane catalyzed by 1

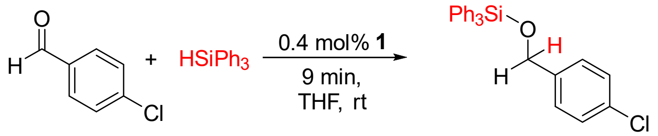

Complex **1** (4.9 mg, 4.8 µmol) was dissolved in THF (6.1 ml) to make a stock solution. To 0.29 ml of this stock solution (0.4 mol% **1**), a mixture of triphenylsilane (1 eq, 15.0 mg, 57.7 µmol) and mesilylene as internal standard (0.4 eq, 22.9 µmol) in THF (0.3 ml) was added. Lastly 4-chlorobenzaldehyde (1 eq, 8.2 mg, 58.3 µmol) was added to the mixture and after 9 min and after 90 minutes, the sample was analyzed by ^{1}H NMR (4 scans, 20 seconds relaxation delay). The product formation was monitored by following the $Ph_3Si\underline{H}$ peak (5.41 ppm), the 4-ClPhC$\underline{H}_2$OSiPh$_3$ (4.83 ppm), the CH_2 of the Tishchenko product (5.28 ppm) and the aromatic mesitylene signal (6.70 ppm) as internal standard to determine the progression of the reaction. 49% conversion and 42% yield was observed after 9 minutes. After 90 min the conversion had reached 80%. We presume that the lack of full conversion is due to catalyst deactivation since full conversion is observed with higher catalyst loadings. When complex **1** is reacted with 4-chlorobenzaldehyde in the absence of a silane, 4-chlorobenzyl 4-chlorobenzoate ester is formed through the Tishchenko reaction. The Tishchenko reaction is catalyzed by strong bases which is why **1** as well as other mono- and double deprotonanted PNNP complexes such as [^{tBu}PNNP**Cu$_2$Mes]K18-crown-6 and **A** all catalyze this reaction. In the presence of silane, the 4-chlorobenzyl 4-chlorobenzoate is not observed since the competing hydrosilylation reaction is faster.

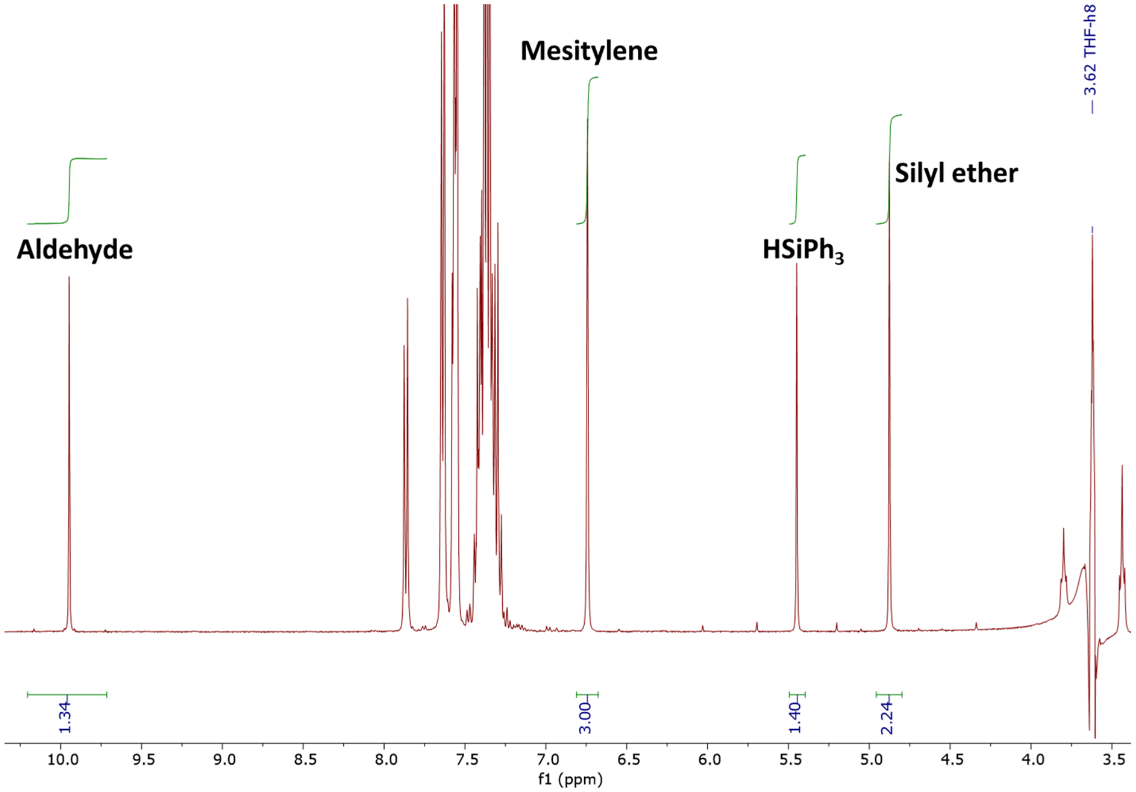

*Figure S21: ^{1}H NMR spectrum after 9 minutes of the hydrosilylation of 4-Chlorobenzaldehyde with triphenylsilane with 0.4 mol% **1** as catalyst in THF-h_8 at 25°C with PRESAT solvent suppression for the THF peaks and 20 seconds relaxation delay.*

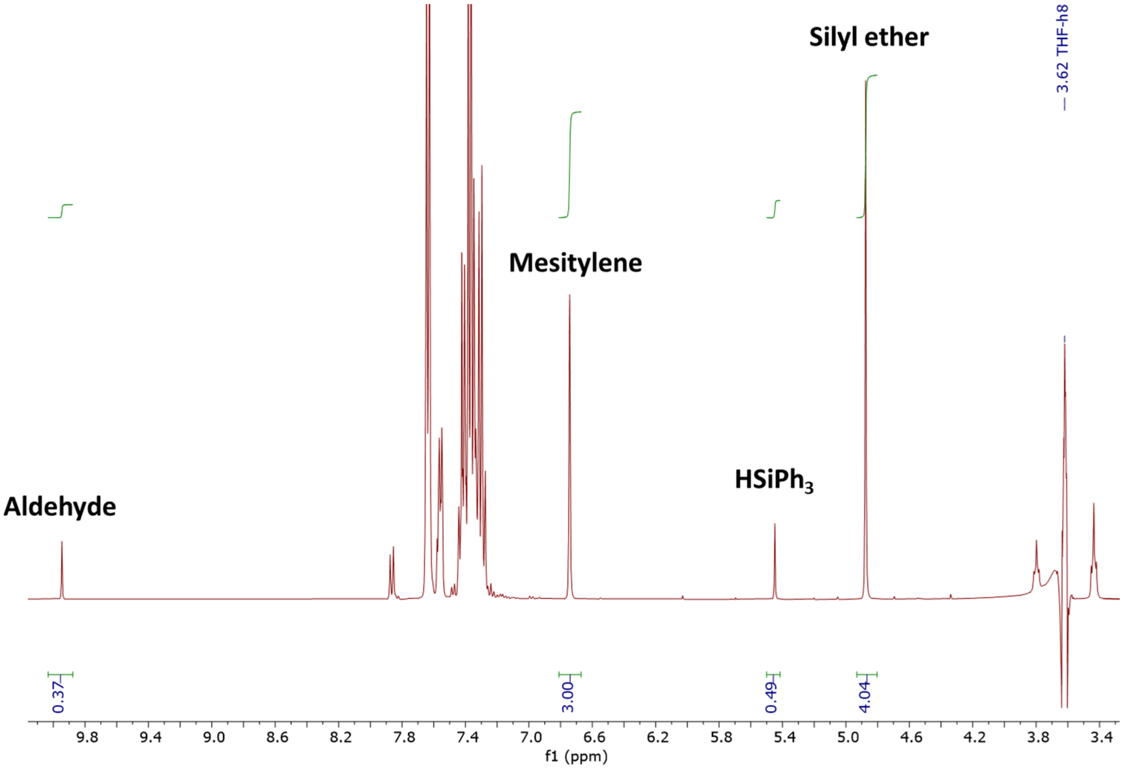

*Figure S22: ^{1}H NMR spectrum after 90 minutes of the hydrosilylation of 4-Chlorobenzaldehyde with triphenylsilane with 0.4 mol% **1** as catalyst in THF-h_8 at 25°C with PRESAT solvent suppression for the THF peaks and 20 seconds relaxation delay.*

Hydrosilylation of benzophenone with diphenylsilane catalyzed by **1**

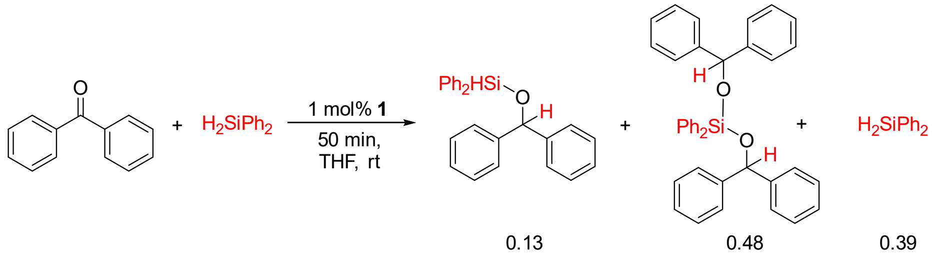

Complex **1** (1 mol%, 1.4 mg, 1.4 µmol) was dissolved in THF (0.5 ml). Benzophenone (1 eq, 25.1 mg, 138 µmol) was dissolved in THF (0.5 ml) and diphenylsilane (1 eq, 25 µl, 135 mmol) was added. The mixture of benzophenone and diphenylsilane was added the solution of **1** and after 50 minutes the mixture was analyzed with NMR.

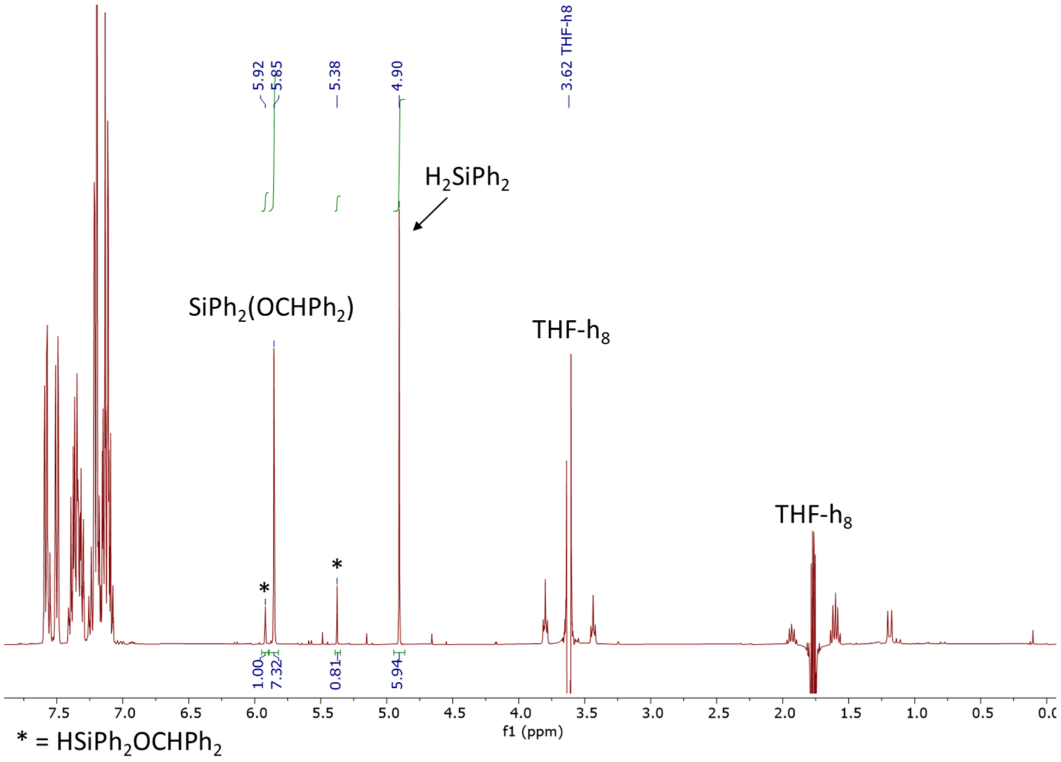

Figure S23: ^{1}H NMR spectrum of the hydrosilylation of benzophenone with triphenylsilane with 1 mol% ***1*** *as catalyst in THF-h_8 at 25°C with PRESAT solvent suppression for the THF peaks and 20 seconds relaxation time.*

Stoichiometric hydrosilylation of benzophenone with diphenylsilane-d_2 catalyzed by **1**

Benzophenone (1.3 eq, 1.1 mg, 6.0 µmol) was dissolved in THF (0.7 ml) this was added to complex **1** (1 eq, 4.8 mg, 1.4 µmol). Then, diphenylsilane-d_2 (1.1 eq, 1 µl, 5.4 µmol) was added to the mixture. After 10 min the mixture was analyzed by ^{1}H NMR (8 scans, 20 seconds relaxation delay), monitoring the $Ph_2C\underline{H}OSiHPh_2$ peak (5.85 ppm) and the $(Ph_2C\underline{H}O)_2SiPh_2$ peak (5.92 ppm) to determine their integral relative to signals of the complex backbone (6.12 and 5.55 ppm). No more benzophenone was visible in the spectrum after the reaction, indicative of full conversion.

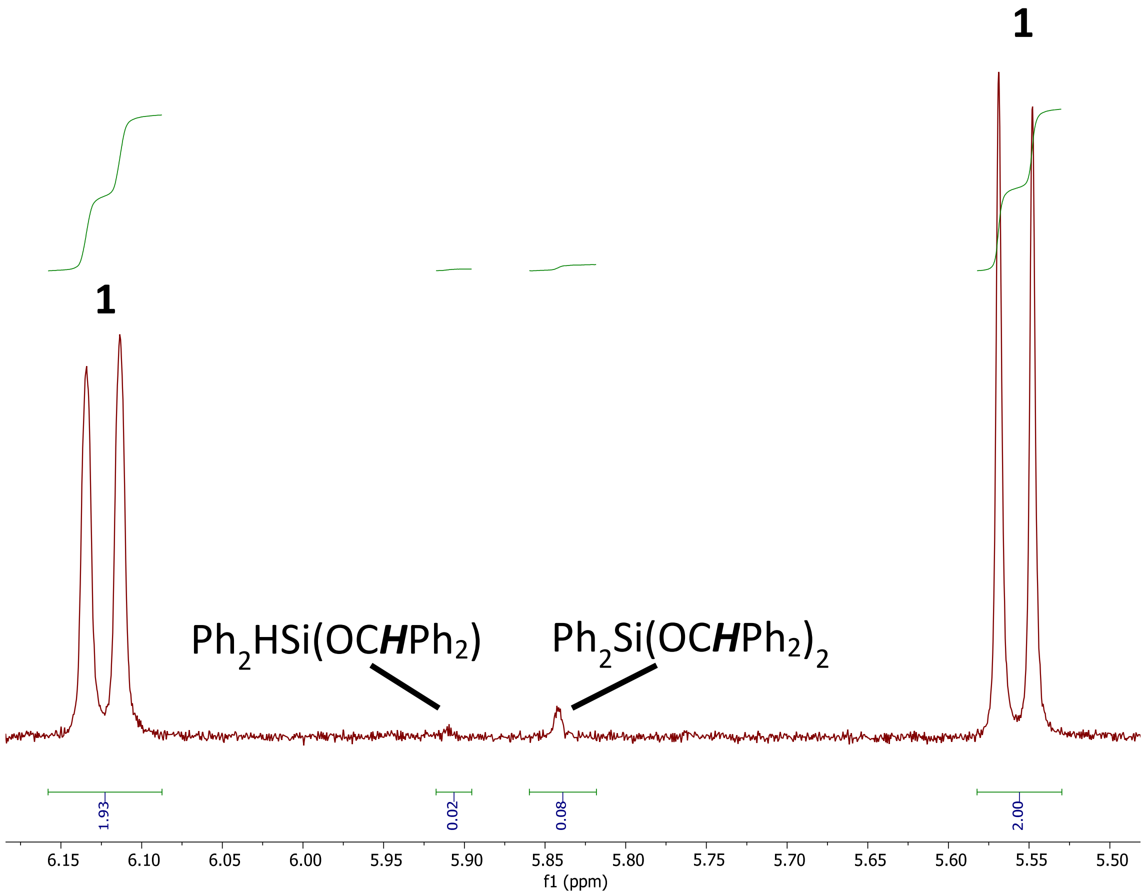

*Figure S24: ^{1}H NMR spectrum of the stoichiometric hydrosilylation of benzophenone with diphenylsilane-d_2 with 1 eq of **1** as catalyst in THF-h_8 at 25°C with PRESAT solvent suppression for the THF peaks.*

Silane scrambling of (diphenylmethoxy)diphenylsilane catalyzed by 1

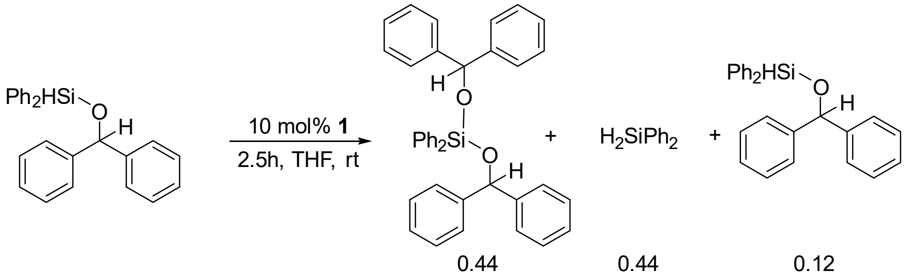

Complex **1** (1 eq, 1.3 mg, 1.3 μmol) was dissolved in THF (0.5 ml) and to this solution, a solution of $SiHPh_2(OCHPh_2)$ in THF (0.5 ml) was added. The mixture was analyzed with ^{1}H NMR after 2,5 hours to determine the ratio between the $Ph_2C\underline{H}OSiHPh_2$ peak (5.85 ppm), the $(Ph_2C\underline{H}O)_2SiPh_2$ peak (5.92 ppm) and the $\underline{H}_2SiPh_2$ peak (4.90 ppm).

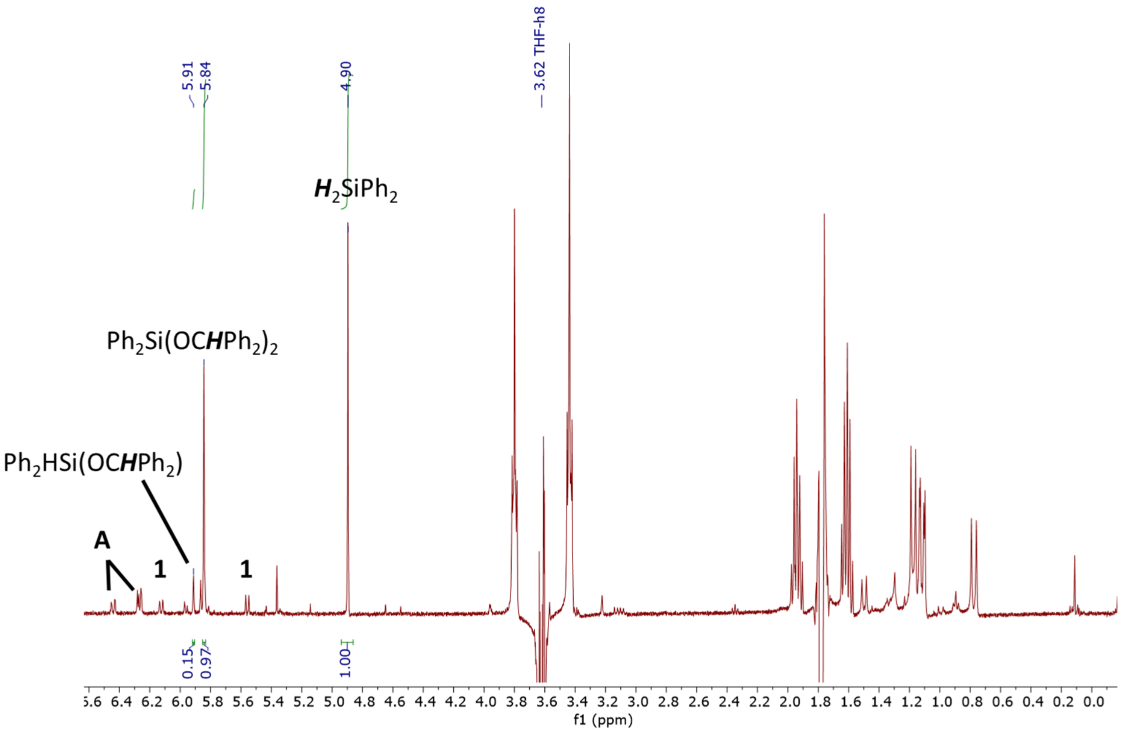

*Figure S25: ^{1}H NMR spectrum of the catalyzed scrambling of $SiHPh_2(OCHPh_2)$ with 10 mol% of **1** as catalyst in THF-h_8 at 25°C with PRESAT solvent suppression for the THF peaks and 20 seconds relaxation time.*

Hydrosilylation of benzophenone with triphenylsilane catalyzed by [PNNP**Cu2Mes]K(18-crown-6)

Triphenylsilane (1 eq, 6.6 mg, 25.3 µmol) was dissolved in THF (0.5 ml) and added to benzophenone (1 eq, 4.6 mg, 25.2 µmol). [PNNP**Cu2Mes]K18-crown-6 (20 mol %, 5.6 mg, 4.9 µmol) was dissolved in THF (0.5 ml) and was added to the mixture with substrates. After 1 hour, the reaction was analyzed by ^{1}H NMR, monitoring the $Ph_3Si\underline{H}$ peak (5.44 ppm), the $Ph_2C\underline{H}OSiPh_3$ (5.97 ppm) and the naphthyridine signal of the complex (6.12 ppm) to determine the progression of the reaction.

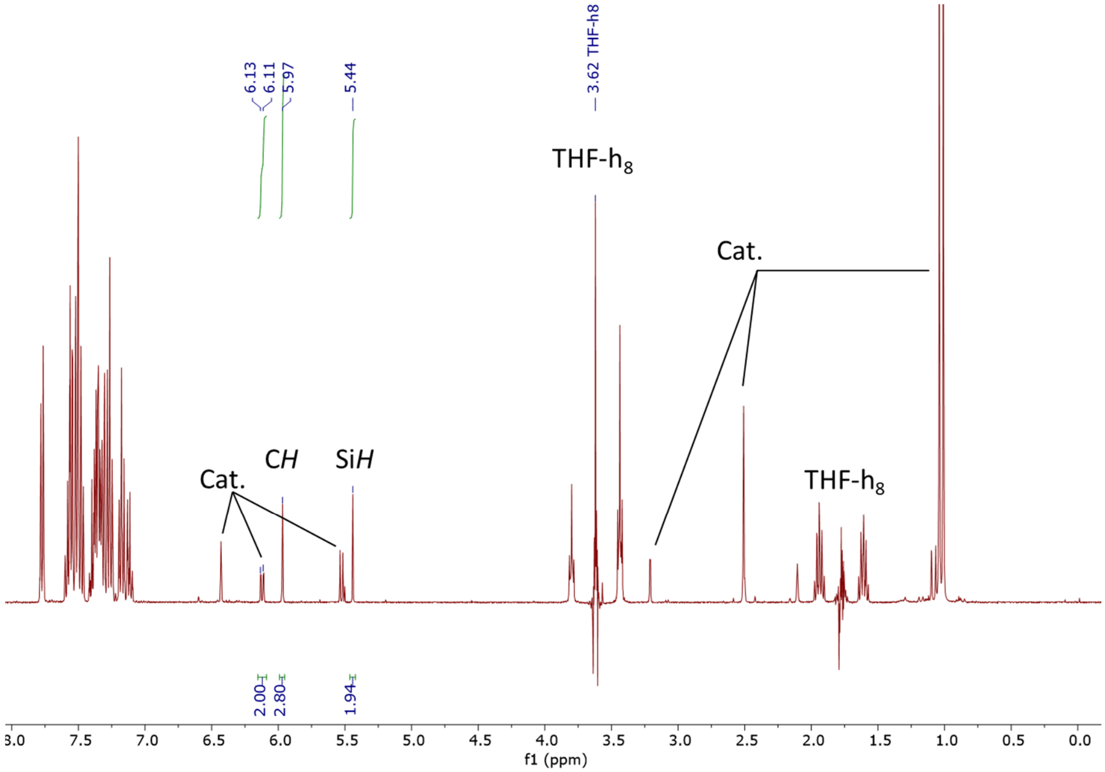

Figure S26: ^{1}H NMR spectrum of the hydrosilylation of benzophenone with triphenylsilane with 20 mol% [PNNP**Cu2Mes]K18-crown-6 as catalyst in THF-h_8 at 25°C with PRESAT solvent suppression for the THF peaks.

Hydrosilylation of benzophenone with triphenylsilane using Stryker's reagent as catalyst

Stryker's reagent (5.6 mg, 2.86 µmol) was dissolved in a mixture of triphenylsilane (1 eq, 15.0 mg, 57.7 µmol) and mesitylene as internal standard (0.4 eq, 22.9 µmol) in THF (0.3 ml). Then, benzophenone (1 eq, 10.5 mg, 57.6 µmol) was added and the reaction was monitored with ^{1}H NMR, showing no product formation after 3 days.

Proposed mechanism for ketone hydrosilylation

From the deuterium labeling experiments it can be inferred that in the mechanism, the C*H* on the former carbonyl position is coming from the silane and not from the hydride in **1**. Hence, if the silane is activated, the hydride from the incoming silane cannot become chemically equivalent to the hydride of **1**. Taking inspiration from the proposals by Bellemin-Laponnaz and coworkers[22], we considered a modified Ojima mechanism and a hypervalent silicon mechanism (Figure S27). The first is the bimetallic Ojima mechanism in which the first step is a bimetallic oxidative addition of the Si–H bond over the two copper centers. Considering the sterics of the formed intermediate of this cis addition (A), it seems unlikely that a carbonyl would insert into the Cu–H bond of the hydride coming from the silane. Therefore we propose that a hypervalent silicon mechanism is most likely for **1** . Variations of this type of mechanism have been proposed for copper and other transition metal based catalysts.[22,23] Also base catalyzed hydrosilylation of ketones are thought to follow this mechanism.[24] The first step (A) of the hypervalent silicon mechanism is the nucleophilic attack of the hydride of **1** on silicon, forming a pentavalent Si intermediate. This is followed by the coordination of the ketone to Si (B) and the insertion of the ketone into the Si–H bond (C). Step D closes the cycle by releasing the siloxane product. At this point we cannot exclude either pathway, but future research is aimed at elucidation of the operational mechanism.

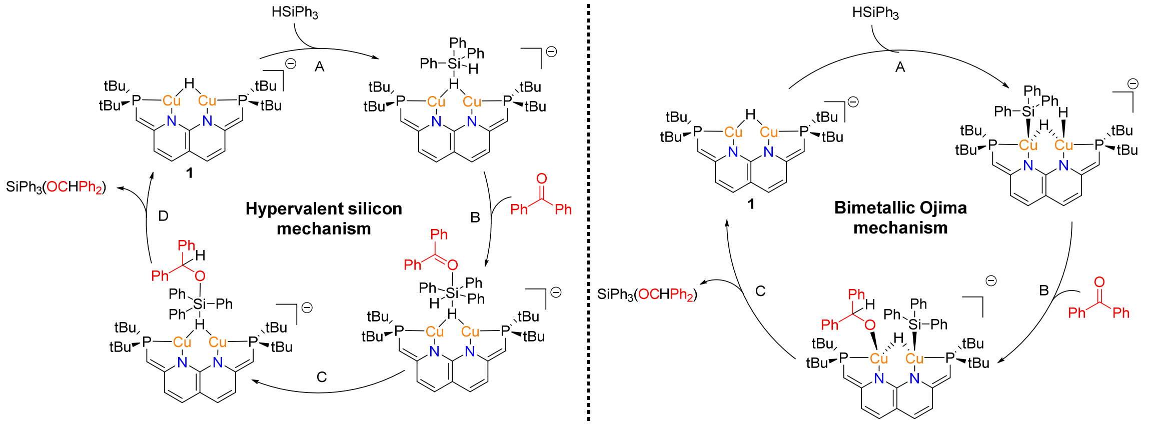

*Figure S27: The proposed hypervalent silicon mechanism (left) and bimetallic Ojima mechamism (right) for hydrosiliation of ketones catalyzed by complex **1**.*

Computational methods

General remarks

Calculations were performed using Gaussian 16 rev. C01 software.[25] The Becke 1988 exchange functional[26] was used in combination with the Perdew 1986 correlation functional(BP86)[27]. The redefinition of Ahlrichs triple-zeta split valence basis set (def2-TZVP) was used on all atoms.[28] No empirical dispersion was employed since this has been shown to provide less accurate results for **A** due to an overestimation of the attractive dispersive energy, which is likely caused by the steric crowding in that molecule.[13,29] NBO[30] calculations were performed using the NBO 6.0 software.[31] Natural population analysis[32,33], Mulliken population analysis[34] and computation of Wiberg bond indices[35] was also performed using the NBO 6.0 software. QTAIM analysis[36] and visualization was performed using the MultiWFN program.[37] Generating QTAIM basins and integration of the electron density in them was performed with MultiWFN using a 'high grid' (with a spacing of 0.06 Bohr). Starting geometries for the optimizations were obtained from the coordinates of the crystal structures if possible, or by modification of the optimized geometry of the most similar complex.

Geometry optimizations

The calculated structure of **1** is a close match in geometry with the crystal structure (figure S28). The Cu–H bond lengths are 1.649 Å, which is the same distance found in complex **A** for the copper centers on the dearomatized side of the naphthyridine pocket.[13] The highest occupied molecular orbital of **1** is located mainly on the backbone (figure S29). The position of the hydride in complex **1** was probed with a relaxed potential energy surface scan to verify that there is not a secondary local minimum corresponding to a terminally bound hydride. The Cu1-Cu2-H34 angle was scanned from its original position (42.5°) in 12 steps of 5°C to its final position of 97.5°. The scan shows a constant increase of energy, confirming the absence of a secondary minimum (Figure S30).

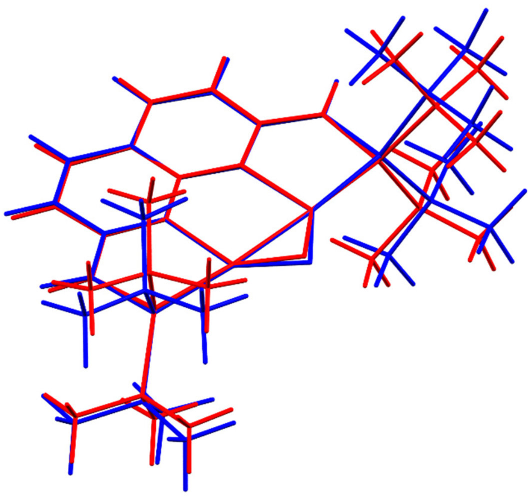

*Figure S28: Overlay of the experimentally determined structure of **1** in the crystal (red) and the computed structure (blue).*

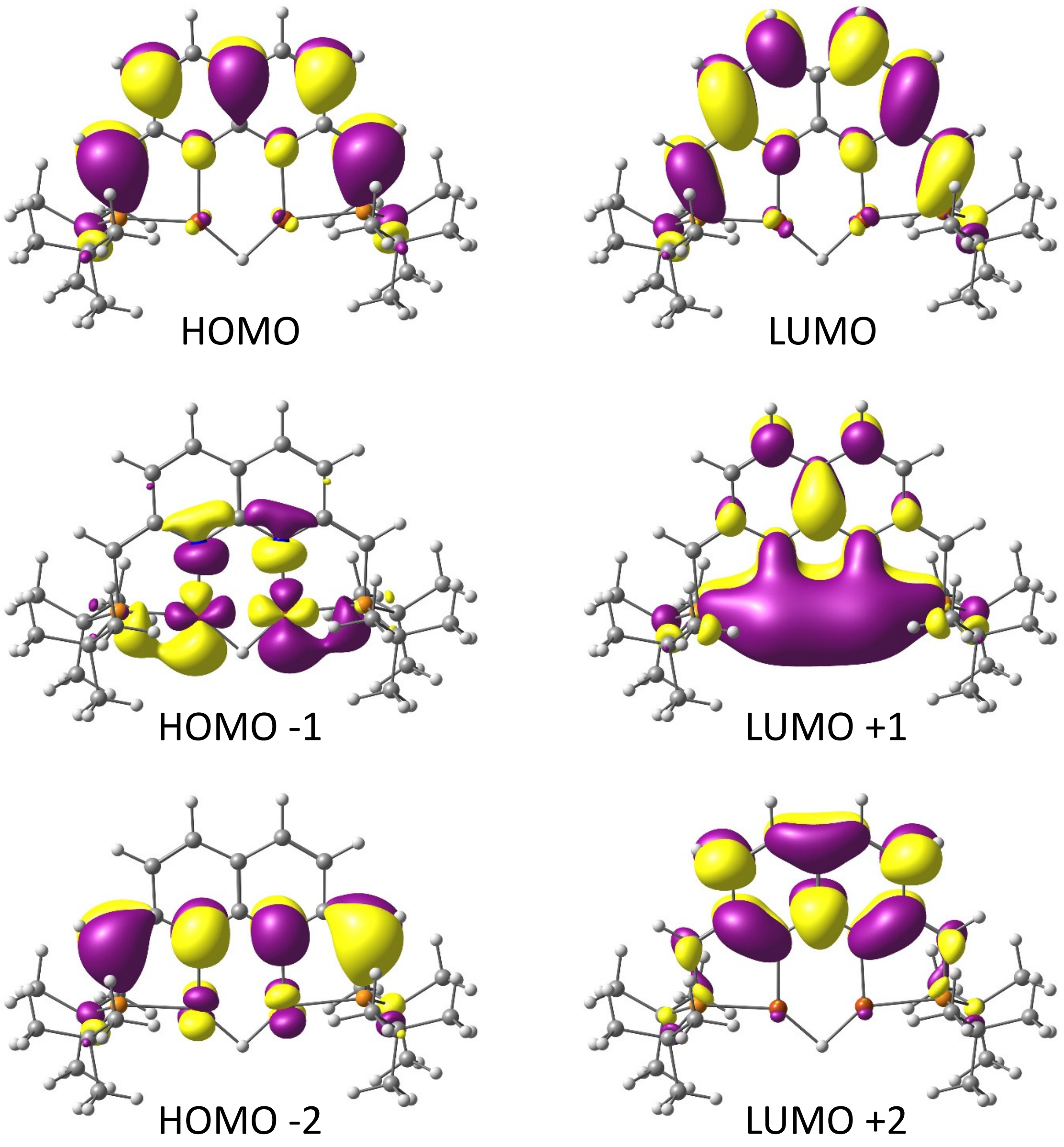

*Figure S29: Visualization of the frontier molecular orbitals of complex **1**.*

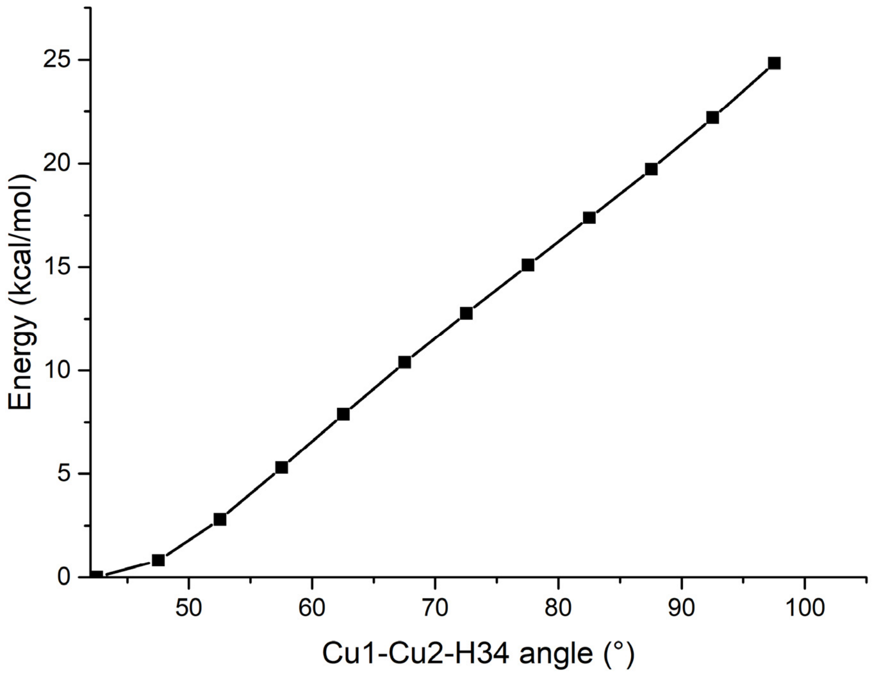

Figure S30: The energy versus the Cu1-Cu2-H34 angle, calculated using a relaxed potential energy surface scan.

Calculated NMR shifts

The ^{1}H shifts of the hydride ligand in **1** where calculated and a predicted chemical shift is 2.67 ppm was found. This is ~1.8 ppm higher than the experimental value. This difference can be ascribed to the lack of relativistic treatment at the level of theory used (BP86/def2-TZVP). When an SMD solvent model (THF) is added, a value of 2.03 ppm is found which is more accurate as expected since solvent effects are known to strongly influence the calculation of ^{1}H shifts. The remaining difference between the experiment and the theoretical calculations can be ascribed to the lack of relativistic treatment at the level of theory used. It has been reported that these relativistic effects are often the reason for the up-field shift of hydrides, hence it fits that a non-relativistic method predicts a shift too downfield.[38] In these calculations the H-P coupling was also probed. Here, a close match with the experiments both with (calc: 24.7 Hz, exp: 25.3 Hz) and without solvent model (calc: 25.8 Hz, exp: 25.3 Hz) is found since the calculation of coupling constants is much less affected by the aforementioned solvent and relativistic effects.

Bonding analysis

Complex 1

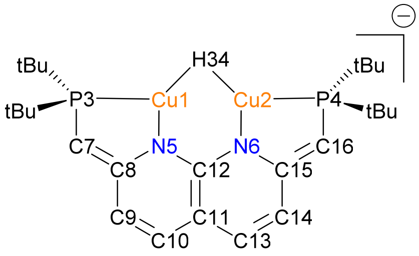

1

NBO analysis assigns a lone-pair on the hydride ligand (H34). This lone-pair delocalizes to both copper centers evenly with a second order perturbation energy of 56.30 kcal/mol each. This is consistent with the C_{2V} symmetry of the complex. There is no donation of copper based orbitals toward the hydride (H34) observed consistent with a 3-center 2-electron bond. The Wiberg bond index between the two copper centers is small (0.1150) consistent with an interaction via the hydride ligand. In the natural binding index, this value is 0.3391, which we propose is also due to the 3-center 2-electron bond in the dicopper hydride core since there is no indication for a direct Cu1-Cu2 interaction in the second order perturbation analysis. This is characteristic for an open 3-center 2-electron bond.[39]

Quantum theory of atoms in molecules (QTAIM) analysis is consistent with the NBO analysis in showing no bond critical point between the two copper centers (Figure S31). As expected, there are bond critical points between the hydride (H34) and the copper centers. These bond critical points are located in the positive area of the Laplacian, indicating a bond with mostly dative character from the hydride to the two copper centers. This is in line with the assignment of an open 3-center 2-electron bond.

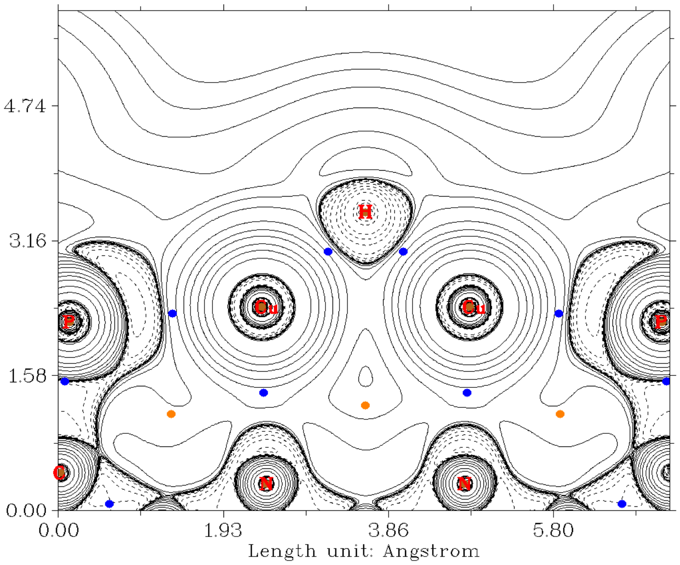

*Figure S31: The Laplacian of the electron density of complex **1** in the Cu–H–Cu plane. Bond critical points are indicated in blue, ring critical points in orange. Solid lines indicate a positive Laplacian and dashed lines a negative one.*

$[IPr_2Cu_2H]BF_4$[4]

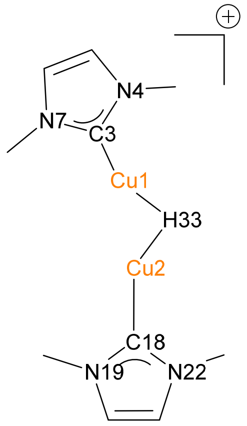

$[IPr_2Cu_2H]BF_4$

For the NBO calculations, the optimized coordinates from the report of Sadighi and co-workers were used.[4] The calculations were performed using the originally reported level of theory (PBEPBE[40]/ZTVP[41,42] with GD2[43] empirical dispersion), as well as the level of theory used for complex **1** (BP86/def2-TZVP). Only the results for the latter will be discussed here since the difference was minimal and does not influence the conclusions.

The hydride (H33) donates towards the copper centers, in this case into the Cu–C anti-bonding orbitals with a second order perturbation energy of 82.41 kcal/mol each. This bonding situation is analogous to what is observed for **1**. Also similarly **1**, a Cu–Cu Wiberg bond index of 0.1100 and a natural binding index of 0.3316 is found for $[IPr_2Cu_2H]BF_4$. This is again consistent with an open type of 3-center 2-electron bond since the second order perturbation analysis shows no indication of direct interaction between the two copper(I) centers. This open type of three center bonding was also already proposed by Sadighi and coworkers based on their calculations.[4] NBO analysis, however, does not indicate the unconventional 3-center 6-electron bond that was originally proposed by the authors.

Hydride charge comparison

To compare the charge of hydride ligand between **1** and **$[IPr_2Cu_2H]BF_4$**, the charges obtained with the natural population analysis were used. To verify that these charges were representative, we decided to also compare the Mulliken charges and the QTAIM charges (obtained by integrating the electron density over the basins) of these two complexes. All three of these methods indicated a more negative charge on the hydride ligand in **1** than in **$[IPr_2Cu_2H]BF_4$** (Table S2).

*Table S2: The charge of the hydride ligand in **1** and **$[IPr_2Cu_2H]BF_4$** calculated using various methods.*

	Natural charge	*Mulliken charge*	*QTAIM charge*
**1**	-0.5011	0.3955	-0.3759
**$[IPr_2Cu_2H]BF_4$**	-0.4250	0.4231	-0.3478

Tracking charge upon deprotonation

To see how the negative charge that is introduced by the deprotonation is distributed, we considered the theoretical scheme in Figure S32. Each of the three structures (**A**, **A_{mono}** and **1**) were optimized and an NBO calculation for each structure was performed. The natural population analysis shows the electron density on each atom, comparing this value between the structures shows the movement of electron density upon deprotonation. Comparing **A** with **A_{mono}** shows the influence of dimerization on the distribution of the charge whereas the comparison between **A_{mono}** and **1** indicates how the negative charge introduced by deprotonation is distributed.

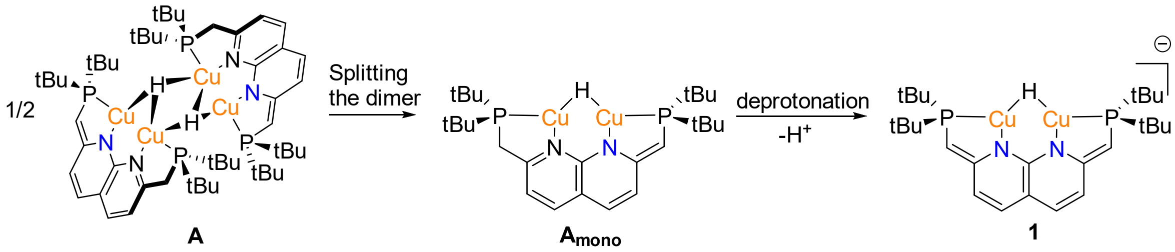

*Figure S32: The deprotonation of **A** to form **1** in two theoretical steps, first is the formation of a monomer and second the deprotonation of this theoretical monomer **A**$_{mono}$ to form **1**.*

In the first step going from **A** to **A**$_{mono}$, the main change in electron density is visible in the core of the molecule (Figure S33). In the core the electron density previously located on the hydride atom (and also to some extend on the phosphorus atoms) is relocated to the two copper centers that have become more electron rich. We rationalize this by considering that the negative hydride is only stabilized by two copper centers in **A**$_{mono}$ rather than three in **A**. This destabilizes the high electron density on the hydride ligand and hence it is found more on the copper centers.

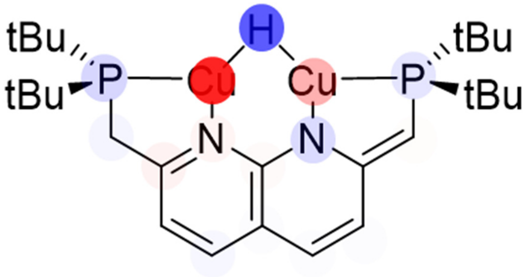

*Figure S33: The change in electron density upon breaking the dimer into monomers (going from **A** to **A**$_{mono}$) calculated using the Natural population analysis. Red indicates an increase in electron density and blue a decrease. The relative amount of change in electron density is indicated by the intensity of the colour (i.e. a darker colour indicates an increase/decrease in electron density).*

When comparing **A**$_{mono}$ to **1** (Figure S34), it becomes clear that the largest part of the introduced negative charge is distributed over the backbone with only a slight (0.02 e^-) increase in the electron density on the hydride ligand. The specific positions in which the electron density increases/decreases can be rationalized by the possible resonance structures of the backbone (Figure S35) Surprisingly, the electron density on the copper centers remains unaffected upon deprotonation.

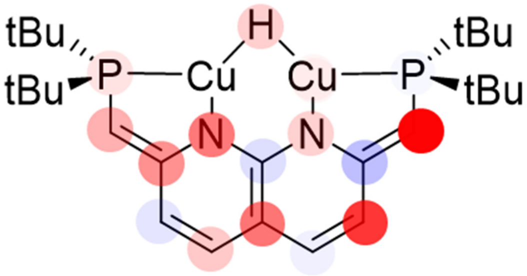

*Figure S34: The change in electron density upon deprotonation (going from **A**$_{mono}$ to **1**) calculated using the Natural population analysis. Red indicates an increase in electron density and blue a decrease. The relative amount of change in electron density is indicated by the intensity of the colour (i.e. a darker colour indicates an increase/decrease in electron density).*

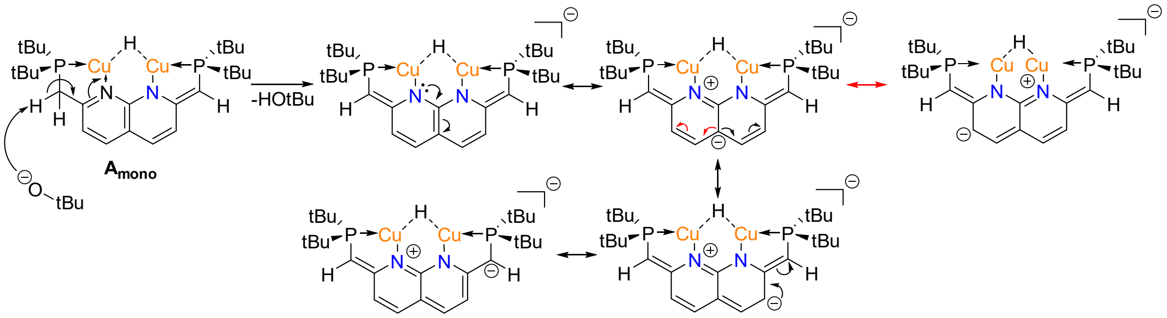

Figure S35: Reaction scheme showing the rationalization for the increase in electron density of certain positions in the backbone upon deprotonation by resonance structures.

Examining the two changes that **A** undergoes upon deprotonation (losing a proton and becoming a monomer) rationalizes the change in electronic structure that is observed. The loss of electron density observed on the hydride despite the addition of a net anionic charge is caused by the formation of a monomer rather than a dimer. This change also causes the increase in electron density on the copper centers. The change in charges on the backbone are mainly due to the deprotonation and delocalization of the negative charge over the conjugated naphthyridine structure.

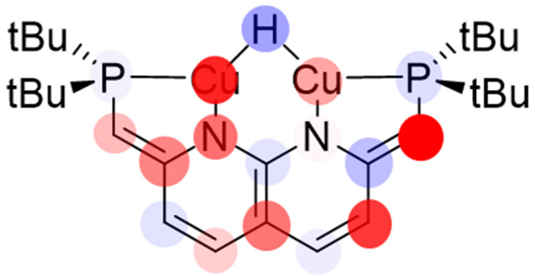

*Figure S36: The change in electron density overall upon deprotonation of **A** (going from **A** to **1**) calculated using the Natural population analysis. Red indicates an increase in electron density and blue a decrease. The relative amount of change in electron density is indicated by the intensity of the colour (i.e. a darker colour indicates an increase in electron density).*

X-ray crystal structure determination

$[C_{20}H_{40}KO_8][C_{26}H_{43}Cu_2N_2P_2] \cdot C_4H_8O$, Fw = 1092.36, orange plate, 0.35 × 0.29 × 0.07 mm^3, monoclinic, $P2_1/c$ (no. 14), a = 9.6631(2), b = 25.2853(6), c = 23.0542(6) Å, β = 90.136(1) °, V = 5632.9(2) $Å^3$, Z = 4, D_x = 1.288 g/cm^3, μ = 0.94 mm^{-1}. The diffraction experiment was performed on a Bruker Kappa ApexII diffractometer with sealed tube and Triumph monochromator (λ = 0.71073 Å) at a temperature of 150(2) K up to a resolution of $(\sin \theta/\lambda)_{max}$ = 0.65 $Å^{-1}$. The Eval15 software[44] was used for the intensity integration. For the prediction of the reflection profiles a split-mosaic model was used. A multi-scan absorption correction and scaling was performed with SADABS[45] (correction range 0.54-0.75). A total of 102303 reflections was measured, 12932 reflections were unique (R_{int} = 0.056), 9412 reflections were observed [$I>2\sigma(I)$]. The structure was solved with Patterson superposition methods using SHELXT.[46] Structure refinement was performed with SHELXL-2018[47] on F^2 of all reflections. Non-hydrogen atoms were refined freely with anisotropic displacement parameters. One coordinated and one non-coordinated THF molecule were refined with disorder models. The metal-coordinated hydride atom was located in difference Fourier maps and kept fixed. All other hydrogen atoms were introduced in calculated positions and refined with a riding model. 699 Parameters were refined with 291 restraints (distances, angles, and displacement parameters in the disordered THF molecules). R1/wR2

[I > 2σ(I)]: 0.0398 / 0.0955. R1/wR2 [all refl.]: 0.0650 / 0.1089. S = 1.020. Residual electron density between -0.34 and 0.53 e/Å^3. Geometry calculations and checking for higher symmetry was performed with the PLATON program.[48]

CCDC 2145253 contains the supplementary crystallographic data for this paper. These data can be obtained free of charge from The Cambridge Crystallographic Data Centre via www.ccdc.cam.ac.uk/data_request/cif.

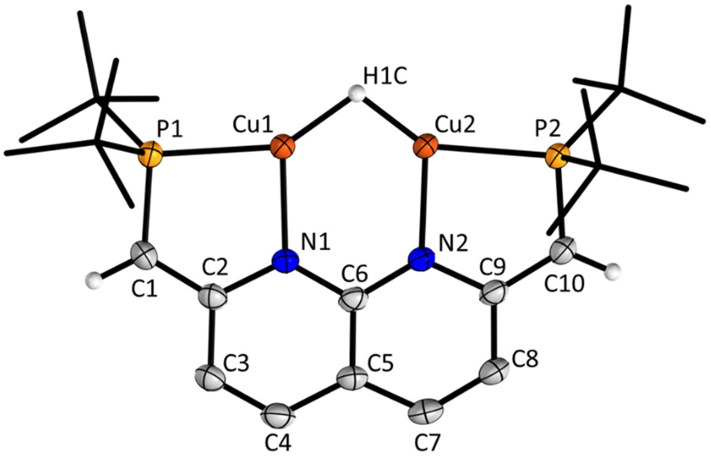

*Figure S37: Anionic moiety of **1** in the crystal, drawn at the 50% probability level. [K((18-crown-6)(THF)$_2$)] cation and disordered THF solvent molecule are omitted for clarity.*

*Table S3: Selected geometrical parameters in the X-ray crystal structure of **1**.*

**Bond lengths**				**Selected angles and torsion angles**	
**Bond**	**Distance [Å]**	**Bond**	**Distance [Å]**	**Atoms**	**Angle [°]**
Cu1-Cu2	2.4230(4)	C2-C3	1.445(3)	Cu1-Cu2-P2	174.67(2)
Cu1-P1	2.2386(6)	C3-C4	1.353(3)	Cu2-Cu1-P1	174.362(19)
Cu2-P2	2.2397(6)	C4-C5	1.415(3)	P1-Cu1-N1	87.12(5)
Cu1-N1	2.0470(18)	C5-C6	1.425(3)	P2-Cu2-N2	87.50(5)
Cu2-N2	2.0422(18)	C6-N1	1.351(3)	N1-Cu1-Cu2	88.52(5)
P1-C1	1.767(2)	C6-N2	1.363(3)	N2-Cu2-Cu1	88.14(5)
P2-C10	1.768(2)	C5-C7	1.413(3)	P1-C1-C2-N1	2.7(3)
C1-C2	1.387(3)	C7-C8	1.355(3)	P2-C10-C9-N2	3.3(3)
C9-C10	1.386(3)	C8-C9	1.447(3)		

References

[1] G. V. Goeden, J. C. Huffman, K. G. Caulton, *Inorg. Chem.* **1986**, *25*, 2484–2485.

[2] N. P. Mankad, D. S. Laitar, J. P. Sadighi, *Organometallics* **2004**, *23*, 3369–3371.

[3] G. D. Frey, B. Donnadieu, M. Soleilhavoup, G. Bertrand, *Chem. - An Asian J.* **2011**, *6*, 402–405.

[4] C. M. Wyss, B. K. Tate, J. Bacsa, T. G. Gray, J. P. Sadighi, *Angew. Chemie - Int. Ed.* **2013**, *52*,

12920–12923.

[5] K. Nakamae, B. Kure, T. Nakajima, Y. Ura, T. Tanase, *Chem. - An Asian J.* **2014**, *9*, 3106–3110.

[6] C. M. Zall, J. C. Linehan, A. M. Appel, *J. Am. Chem. Soc.* **2016**, *138*, 9968–9977.

[7] S. Zhang, H. Fallah, E. J. Gardner, S. Kundu, J. A. Bertke, T. R. Cundari, T. H. Warren, *Angew. Chemie - Int. Ed.* **2016**, *55*, 9927–9931.

[8] E. A. Romero, P. M. Olsen, R. Jazzar, M. Soleilhavoup, M. Gembicky, G. Bertrand, *Angew. Chemie - Int. Ed.* **2017**, *56*, 4024–4027.

[9] A. J. Jordan, C. M. Wyss, J. Bacsa, J. P. Sadighi, *Organometallics* **2016**, *35*, 613–616.

[10] B. L. Tran, B. D. Neisen, A. L. Speelman, T. Gunasekara, E. S. Wiedner, R. M. Bullock, *Angew. Chemie Int. Ed.* **2020**, *59*, 8645–8653.

[11] A. L. Speelman, B. L. Tran, J. D. Erickson, M. Vasiliu, D. A. Dixon, R. M. Bullock, *Chem. Sci.* **2021**, *12*, 11495–11505.

[12] C. R. Groom, I. J. Bruno, M. P. Lightfoot, S. C. Ward, *Acta Crystallogr. Sect. B Struct. Sci. Cryst. Eng. Mater.* **2016**, *72*, 171–179.

[13] E. Kounalis, M. Lutz, D. L. J. Broere, *Chem. – A Eur. J.* **2019**, 13280–13284.

[14] E. Kounalis, M. Lutz, D. L. J. Broere, *Organometallics* **2020**, *39*, 585–592.

[15] M. Brookhart, B. Grant, A. F. Volpe, *Organometallics* **1992**, *11*, 3920–3922.

[16] G. R. Fulmer, A. J. M. Miller, N. H. Sherden, H. E. Gottlieb, A. Nudelman, B. M. Stoltz, J. E. Bercaw, K. I. Goldberg, *Organometallics* **2010**, *29*, 2176–2179.

[17] M. Yasuda, Y. Onishi, M. Ueba, T. Miyai, A. Baba, *J. Org. Chem.* **2001**, *66*, 7741–7744.

[18] W. Sattler, S. Ruccolo, M. Rostami Chaijan, T. Nasr Allah, G. Parkin, *Organometallics* **2015**, *34*, 4717–4731.

[19] M. Feller, U. Gellrich, A. Anaby, Y. Diskin-Posner, D. Milstein, *J. Am. Chem. Soc.* **2016**, *138*, 6445–6454.

[20] M. Vogt, A. Nerush, Y. Diskin-Posner, Y. Ben-David, D. Milstein, *Chem. Sci.* **2014**, *5*, 2043–2051.

[21] O. Rivada-Wheelaghan, A. Dauth, G. Leitus, Y. Diskin-Posner, D. Milstein, *Inorg. Chem.* **2015**, *54*, 4526–4538.

[22] J. T. Issenhuth, S. Dagorne, S. Bellemin-Laponnaz, *Adv. Synth. Catal.* **2006**, *348*, 1991–1994.

[23] M. C. Lipke, A. L. Liberman-Martin, T. D. Tilley, *Angew. Chemie - Int. Ed.* **2017**, *56*, 2260–2294.

[24] K. Revunova, G. I. Nikonov, *Chem. - A Eur. J.* **2014**, *20*, 839–845.

[25] Gaussian 16, Revision C.01, M. J. Frisch, G. W. Trucks, H. B. Schlegel, G. E. Scuseria, M. A. Robb, J. R. Cheeseman, G. Scalmani, V. Barone, G. A. Petersson, H. Nakatsuji, X. Li, M. Caricato, A. V. Marenich, J. Bloino, B. G. Janesko, R. Gomperts, B. Mennucci, H. P. Hratchian, J. V. Ortiz, A. F. Izmaylov, J. L. Sonnenberg, D. Williams-Young, F. Ding, F. Lipparini, F. Egidi, J. Goings, B. Peng, A. Petrone, T. Henderson, D. Ranasinghe, V. G. Zakrzewski, J. Gao, N. Rega, G. Zheng, W. Liang, M. Hada, M. Ehara, K. Toyota, R. Fukuda, J. Hasegawa, M. Ishida, T. Nakajima, Y. Honda, O. Kitao, H. Nakai, T. Vreven, K. Throssell, J. A. Montgomery, Jr., J. E. Peralta, F. Ogliaro, M. J. Bearpark, J. J. Heyd, E. N. Brothers, K. N. Kudin, V. N. Staroverov, T. A. Keith, R. Kobayashi, J. Normand, K. Raghavachari, A. P. Rendell, J. C. Burant, S. S. Iyengar, J.

Tomasi, M. Cossi, J. M. Millam, M. Klene, C. Adamo, R. Cammi, J. W. Ochterski, R. L. Martin, K. Morokuma, O. Farkas, J. B. Foresman, and D. J. Fox, Gaussian, Inc., Wallingford CT, 2019.

[26] A. D. Becke, *Phys. Rev. A* **1988**, *38*, 3098–3100.

[27] J. P. Perdew, *Phys. Rev. B* **1986**, *33*, 8822–8824.

[28] F. Weigend, R. Ahlrichs, *Phys. Chem. Chem. Phys.* **2005**, *7*, 3297.

[29] S. Grimme, J. Antony, S. Ehrlich, H. Krieg, *J. Chem. Phys.* **2010**, *132*, 154104.

[30] J. P. Foster, F. Weinhold, *J. Am. Chem. Soc.* **1980**, *102*, 7211–7218.

[31] NBO 6.0. E. D. Glendening, J. K. Badenhoop, A. E. Reed, J. E. Carpenter, J. A. Bohmann, C. M. Morales, C. R. Landis, and F. Weinhold (Theoretical Chemistry Institute, University of Wisconsin, Madison, WI, 2013)

[32] A. E. Reed, F. Weinhold, *J. Chem. Phys.* **1983**, *78*, 4066–4073.

[33] A. E. Reed, R. B. Weinstock, F. Weinhold, *J. Chem. Phys.* **1985**, *83*, 735–746.

[34] R. S. Mulliken, *J. Chem. Phys.* **1955**, *23*, 1833–1840.

[35] K. B. Wiberg, *Tetrahedron* **1968**, *24*, 1083–1096.

[36] R. F. W. Bader, *Acc. Chem. Res.* **1985**, *18*, 9–15.

[37] T. Lu, F. Chen, *J. Comput. Chem.* **2012**, *33*, 580–592.

[38] A. C. Castro, D. Balcells, M. Repisky, T. Helgaker, M. Cascella, *Inorg. Chem.* **2020**, *59*, 17509–17518.

[39] M. H. Baik, R. A. Friesner, G. Parkin, *Polyhedron* **2004**, *23*, 2879–2900.

[40] J. P. Perdew, K. Burke, M. Ernzerhof, *Phys. Rev. Lett.* **1996**, *77*, 3865–3868.

[41] A. Schäfer, H. Horn, R. Ahlrichs, *J. Chem. Phys.* **1992**, *97*, 2571–2577.

[42] A. Schäfer, C. Huber, R. Ahlrichs, *J. Chem. Phys.* **1994**, *100*, 5829–5835.

[43] S. Grimme, *J. Comput. Chem.* **2006**, *27*, 1787–1799.

[44] A. M. M. Schreurs, X. Xian, L. M. J. Kroon-Batenburg, *J. Appl. Crystallogr.* **2010**, *43*, 70–82.

[45] G. M. Sheldrick, **2014**, SADABS and TWINABS. Universität Göttingen, Germany

[46] G. M. Sheldrick, *Acta Crystallogr. Sect. A Found. Crystallogr.* **2015**, *71*, 3–8.

[47] G. M. Sheldrick, *Acta Crystallogr. Sect. C Struct. Chem.* **2015**, *71*, 3–8.

[48] A. L. Spek, *Acta Crystallogr. Sect. D Biol. Crystallogr.* **2009**, *65*, 148–155.
